# Health workers’ perspectives on the quality of maternal and newborn health care around the time of childbirth: Results of the Improving MAternal Newborn carE in the EURO Region (IMAgiNE EURO) project in 12 countries of the World Health Organization European Region

**DOI:** 10.7189/jogh.14.04164

**Published:** 2024-09-06

**Authors:** Emanuelle Pessa Valente, Ilaria Mariani, Arianna Bomben, Sandra Morano, Michael Gemperle, Marina Ruxandra Otelea, Céline Miani, Helen Elden, Antigoni Sarantaki, Raquel Costa, Barbara Baranowska, Martina König-Bachmann, Sigrun Kongslien, Daniela Drandić, Virginie Rozée, Antonella Nespoli, Alessia Abderhalden-Zellweger, Ioana Nanu, Stephanie Batram-Zantvoort, Karolina Linden, Dimitra Metallinou, Heloísa Dias, Urszula Tataj-Puzyna, Elisabeth D’Costa, Ingvild Hersoug Nedberg, Magdalena Kurbanović, Elise de La Rochebrochard, Simona Fumagalli, Susanne Grylka-Baeschlin, Claudia Mariana Handra, Mehreen Zaigham, Eirini Orovou, Catarina Barata, Beata Szlendak, Christoph Zenzmaier, Eline Skirnisdottir Vik, Alina Liepinaitienė, Zalka Drglin, Maryse Arendt, Emma Sacks, Marzia Lazzerini, Improving MAternal Newborn carE in the EURO Region (IMAgiNE EURO) Study Group, Martina König-Bachmann, Christoph Zenzmaier, Simon Imola, Elisabeth D’Costa, Anne Galle, Silke D’Hauwers, Amira Ćerimagić, Ourania Kolokotroni, Eleni Hadjigeorgiou, Maria Karanikola, Nicos Middleton, Ioli Orphanide, Daniela Drandić, Magdalena Kurbanović, Lenka Laubrova Zirovnicka, Miloslava Kramná, Rozée Virginie, Elise de La Rochebrochard, Kristina Löfgren, Céline Miani, Stephanie Batram-Zantvoort, Antigoni Sarantaki, Dimitra Metallinou, Aikaterini Lykeridou, Eirini Orovou, Ilana Chertok, Rada Artzi-Medvedik, Marzia Lazzerini, Emanuelle Pessa Valente, Ilaria Mariani, Arianna Bomben, Stefano Delle Vedove, Sandra Morano, Antonella Nespoli, Simona Fumagalli, Elizabete Pumpure, Dace Rezeberga, Dārta Jakovicka, Gita Jansone-Šantare, Anna Šibalova, Elīna Voitehoviča, Dārta Krēsliņa, Alina Liepinaitienė, Andželika Kondrakova, Marija Mizgaitienė, Simona Juciūtė, Maryse Arendt, Barbara Tasch, Enrico Lopriore, Thomas Van den Akker, Ingvild Hersoug Nedberg, Sigrun Kongslien, Eline Skirnisdottir Vik, Barbara Baranowska, Urszula Tataj-Puzyna, Beata Szlendak, Paulina Pawlicka, Raquel Costa, Catarina Barata, Teresa Santos, Heloísa Dias, Tiago Miguel Pinto, Sofia Marques, Ana Meireles, Joana Oliveira, Mariana Pereira, Maria Arminda Nunes, Marina Ruxandra Otelea, Jelena Radetić, Jovana Ružičić, Zalka Drglin, Anja Bohinec, Serena Brigidi, Alejandra Oliden, Lara Martín Castañeda, Helen Elden, Region Västra Götaland, Karolina Linden, Mehreen Zaigham, Claire de Labrusse, Alessia Abderhalden-Zellweger, Anouck Pfund, Harriet Thorn, Susanne Grylka, Michael Gemperle, Antonia Mueller

**Affiliations:** 1 World Health Organization Collaborating Centre for Maternal and Child Health, Institute for Maternal and Child Health IRCCS Burlo Garofolo, Trieste, Italy; 2 Medical School and Midwifery School, Genoa University, Genoa, Italy; 3 Institute of Midwifery and Reproductive Health, School of Health Professions, ZHAW Zurich University of Applied Sciences, Winterthur, Switzerland; 4 University of Medicine and Pharmacy Carol Davila, Bucharest, Romania; 5 SAMAS Association, Bucharest, Romania; 6 Department of Epidemiology and International Public Health, School of Public Health, Bielefeld University, Bielefeld, Germany; 7 Institute of Health and Care Sciences, Sahlgrenska Academy, University of Gothenburg, Gothenburg, Sweden; 8 Department of Obstetrics and Gynaecology, Region Västra Götaland, Sahlgrenska University Hospital, Gothenburg, Sweden; 9 Department of Midwifery, Faculty of Health and Care Sciences, University of West Attica, Athens, Greece; 10 EPIUnit, Instituto de Saúde Pública, Universidade do Porto, Porto, Portugal; 11 Laboratório para a Investigação Integrativa e Translacional em Saúde Populacional, Porto, Portugal; 12 Lusófona University, HEI-Lab: Digital Human-Environment Interaction Labs, Lisboa, Portugal; 13 Department of Midwifery, Centre of Postgraduate Medical Education, Warsaw, Poland; 14 Health University of Applied Sciences Tyrol, Innsbruck, Austria; 15 Department of Health and Care Sciences, UiT The Arctic University of Norway, Norway; 16 International Confederation of Midwives (ICM), Hague, Netherlands; 17 Roda – Parents in Action, Zagreb, Croatia; 18 Sexual and Reproductive Health and Rights Research Unit, Institut National d’Études Démographiques, Paris, France; 19 School of Medicine and Surgery, University of Milano-Bicocca, Monza, Italy; 20 Department of Obstetrics, Foundation IRCCS San Gerardo dei Tintori Monza, Italy; 21 School of Health Sciences (HESAV), HES-SO University of Applied Sciences and Arts Western Switzerland, Lausanne, Switzerland; 22 Social Obstetrics and Paediatric Research Unit, National Institute for Mother and Child Health Alessandrescu Rusescu, Bucharest, Romania; 23 Regional Health Administration of the Algarve, IP (ARS - Algarve), Portugal; 24 Medical University of Innsbruck, Innsbruck, Austria; 25 Faculty of Health Studies, University of Rijeka, Rijeka, Croatia; 26 Obstetrics and Gynaecology, Department of Obstetrics and Gynaecology, Institution of Clinical Sciences Lund, Lund University, Lund and Skane University Hospital, Malmö, Sweden; 27 Department of Midwifery, School of Health Sciences, University of Western Macedonia, Ptolemaida, Greece; 28 Instituto de Ciências Sociais, Universidade de Lisboa, Lisboa, Portugal; 29 Associação Portuguesa Pelos Direitos da Mulher na Gravidez e Parto, Lisbon, Portugal; 30 Department of Health and Caring Sciences, Western Norway University of Applied Sciences, Norway; 31 Faculty of Natural Sciences, Department of Environmental Sciences, Vytautas Magnus University, Kaunas, Lithuania; 32 Faculty of Medicine, Kauno Kolegija Higher Education Institution, Kaunas, Lithuania; 33 Republican Siauliai County Hospital, Siauliai, Lithuania; 34 National Institute of Public Health, Ljubljana, Slovenia; 35 Professional association of the Lactation Consultants in Luxembourg, Luxembourg, Luxembourg; 36 Department of International Health, Johns Hopkins University, Baltimore, Maryland, USA; 37 London School of Hygiene and Tropical Medicine, London, UK

## Abstract

**Background:**

Health workers’ (HWs’) perspectives on the quality of maternal and newborn care (QMNC) are not routinely collected. In this cross-sectional study, we aimed to document HWs' perspectives on QMNC around childbirth in 12 World Health Organization (WHO) European countries.

**Methods:**

HWs involved in maternal/neonatal care for at least one year between March 2020 and March 2023 answered an online validated WHO standards-based questionnaire collecting 40 quality measures for improving QMNC. A QMNC index (score 0–400) was calculated as a synthetic measure.

**Results:**

Data from 4143 respondents were analysed. For 39 out of 40 quality measures, at least 20% of HWs reported a ‘need for improvement’, with large variations across countries. Effective training on healthy women/newborns management (n = 2748, 66.3%), availability of informed consent job aids (n = 2770, 66.9%), and effective training on women/newborns rights (n = 2714, 65.5%) presented the highest proportion of HWs stating ‘need for improvement’. Overall, 64.8% (n = 2684) of respondents declared that HWs’ numbers were insufficient for appropriate care (66.3% in Portugal and 86.6% in Poland), and 22.4% described staff censorship (16.3% in Germany and 56.7% in Poland). The reported QMNC index was low in all countries (Poland median (MD) = 210.60, interquartile range (IQR) = 155.71, 273.57; Norway MD = 277.86; IQR = 244.32, 308.30). The ‘experience of care’ domain presented in eight countries had significantly lower scores than the other domains (*P* < 0.001). Over time, there was a significant monthly linear decrease in the QMNC index (*P* < 0.001), lacking correlation with the coronavirus disease 2019 (COVID-19) pandemic trends (*P* > 0.05). Multivariate analyses confirmed large QMNC variation by country. HWs with <10 years of experience, HWs from public facilities, and midwives rated QMNC with significantly lower scores (*P* < 0.001).

**Conclusions:**

HWs from 12 European countries reported significant gaps in QMNC, lacking association with COVID-19 pandemic trends. Routine monitoring of QMNC and tailored actions are needed to improve health services for the benefit of both users and providers.

**Registration:**

ClinicalTrials.gov NCT04847336.

It is increasingly recognised that the quality of maternal and newborn care (QMNC) is a major determinant of maternal and newborn health outcomes and health services costs [1,2]. Regrettably, reaching high-quality health systems remains a global challenge, even in high-income countries. Disrespect and abuse, non-evidence-based practices and over-medicalisation during childbirth have been frequently reported and observed [3–10]. Inadequate planning and management of the maternal and newborn health workforce and a lack of initiatives that value the importance of health workers (HWs) have been identified as key reasons for the low quality of care [11–14].

Since 2016, the World Health Organization (WHO) standards for improving the QMNC [15] define a set of quality measures divided into three key domains – provision of care, experience of care, and availability of competent and motivated human and physical resources – that can be used to monitor, assess, and improve QMNC at facility level. Both health service providers and users can yield different and useful information on those measures from different perspectives. HWs views on the QMNC are not routinely collected and have rarely been reported by research studies or used to enhance the monitoring of key indicators in low- or high-income countries [16–20].

The Improving MAternal Newborn carE in the EURO Region (IMAgiNE EURO) project utilised two online, anonymous, validated questionnaires based on the WHO standards [15], each including 40 key WHO quality measures, for collecting two complementary perspectives on the QMNC – the perspective of women [21], and of HWs [22]. Several previous publications of IMAgiNE EURO data documented significant gaps in the QMNC from women’s perspectives, with large systematic inequalities across and within WHO European countries [23–30]. Other researchers have described the importance of triangulating women’s and HW’s perspectives to identify adequate actions for improving QMNC, including in demanding events such as pandemics [31–40]. However, few quantitative data are available from the HWs’ perspective on QMNC in the WHO European Region important quality gaps [17,35,37]. In particular, when using the WHO standards [15] as reference [35,37], there is a lack of studies allowing over time and across countries comparisons of relevant domains to QMNC (i.e. provision of care, experience of care, availability of competent and motivated human and physical resources and organisational changes due to the coronavirus disease 2019 (COVID-19) pandemic). As part of the IMAgiNE EURO project, in this paper, we aimed to document HWs perspectives on the QMNC around the time of childbirth at the facility level in 12 countries of the WHO Europe Region in the period between 2020–23, as measured by 40 WHO-standard-based quality measures and by a QMNC index (total and by the four QMNC domains). We also investigated how the total QMNC index changed over time compared to the COVID-19 pandemic trends.

## METHODS

### Study design and participants

This cross-sectional study is reported according to the Strengthening the Reporting of Observational Studies in Epidemiology (STROBE) [41] (Table S1 in the **Online Supplementary Document**).

We invited to participate HWs who were directly involved in maternal/neonatal care at the facility level (i.e. general physicians currently working in maternal or neonatal care, midwives, nurses, neonatologists, obstetrician gynaecologists (OBGYN) doctors, and medical residents in OBGYN or neonatology) for at least one year between 1 March 2020 and 1 March 2023. HWs not matching the above inclusion criteria or not working in a country in the WHO Europe Region were excluded.

### Data collection

We collected data using a validated, self-administrated, anonymous online questionnaire [22] based on WHO standards [15]. IMAgiNE EURO project partners actively promoted the survey following setting-specific dissemination plans decided by local research groups in each participating country. Dissemination plans targeted HWs directly involved in maternal/neonatal care at the facility level. The main approaches included: link to survey shared by dissemination on social media (e.g. Facebook, Instagram, X (formerly known as Twitter), and LinkedIn), institutional websites and newsletters, study presentations for hospitals’ staff and during national scientific conferences, diffusion by institutional mailing lists of national HWs professional associations, local professional or personal networks, and nongovernmental organisations. We collected data using REDCap, version 8.5.21(Vanderbilt University, Nashville, Tennessee, USA), as a centralised platform.

Details on questionnaire development, validation, translation and cultural adaptation have been reported elsewhere [22]. Briefly, the validation process included content, construct, and face validity assessed through a Delphi study among a multidisciplinary group of experts and assessment of internal consistency, interrater reliability, and acceptability (Figure S1 in the **Online Supplementary Document**). The survey was then translated and back-translated following the guidance of the Professional Society for Health Economics and Outcomes Research Task Force for Translation and Cultural Adaptation Principles of Good Practice [42]. The survey was made available in 17 languages (Table S2 in the **Online Supplementary Document**), and HWs were invited to answer in their preferred language, regardless of their country of work.

The questionnaire included two parallel pathways, one for HWs providing maternal care and one for HWs providing neonatal care [22]. These differed only by three questions, of which two related to training and one to the right of informed choice on specific procedures related either to maternal or neonatal health. HWs providing both maternal and neonatal care – such as midwives – could answer questions related to both pathways. Each pathway consisted of 40 WHO standards-based quality measures [15], 10 for each of the four domains of the questionnaire: 1) provision of care, 2) experience of care, 3) availability of motivated and competent human and physical resources and an additional domain, 4) organisational changes due to COVID-19 pandemic. It also included 13 sociodemographic questions placed at the end of the questionnaire [22].

The 40 quality measures contributed to a QMNC index [22] (Table S3 in the **Online Supplementary Document**), which was developed drawing on previous examples [23,43] as a synthetic and complementary measure of QMNC. A score ranging from 0–10 was attributed to each question. Consequently, for each of the four domains, the score ranged from 0–100; their sum for the four domains constituted the total QMNC index, ranging from 0–400, with higher scores indicating higher adherence to WHO standards [15]. For HWs providing maternal and neonatal care, an index score was calculated for each pathway, and the mean value was retained as the total QMNC index.

### Statistical analyses

A sample size of 100 HW was needed for each country based on the hypothesis of an average QMNC index (our primary outcome) of 75% (margin of error (MOE) = 8.5) (300 points (MOE = 34), out of 400 points) and confidence level of 95%. This sample was also adequate to detect a minimum frequency variation on each quality measure of 4% (MOE = 4), with a confidence level of 96%. The upper limit of the sample was not predefined.

We considered as not informative unfinished records with 36 or more (≥90%) missing quality measures and we excluded these from primary analysis, in line with other similar studies [17]. We classified two or more questionnaires completed within an hour on the same day as suspected duplicates when answers to sociodemographic variables, quality measures, geographic region (where available), and language of questionnaire completion were the same. Suspected duplicates were excluded, and only the most recent entry was kept for analysis.

Questionnaires with the same pattern of answers for all quality measures (i.e. always answer option one (high quality of care) or always answer option three (inappropriate quality of care)) resulting in extreme values for the QMNC index were also excluded. We assumed that individuals responding to the questionnaire attentively would not have used the same pattern of responses throughout the whole questionnaire [44] while including even a low proportion of these outliers would have impacted results substantially [45,46].

We calculated overall sample characteristics and frequencies for each quality measure for each country, with a sample size of more than 100 respondents. Quality measures were presented according to the domain of the WHO framework, based on which the WHO standards were developed [15].

For the 30 quality measures in the domains of provision of care, experience of care, and availability of competent and motivated human and physical resources, three answers were possible when HWs assessed quality measures: ‘yes, it was available/adequate’; ‘it needs some improvement’; and ‘it needs significant improvement’. For the primary analysis, we presented the combined frequency of the two answers, ‘it needs some improvement’ and ‘it needs significant improvement’. To further assess findings, as secondary analysis, we also separately analysed the frequency of the answer ‘it needs significant improvement’.

The domain of organisational changes due to the COVID-19 pandemic had two possible groups of answers according to the indicator type. The first group of possible answers was: ‘existing and/or adequate since the beginning of the pandemic’; ‘not always existing and/or not fully adequate’, e.g. lacking in the first phase of the pandemic; and ‘never existed and/or never adequate since the beginning of pandemic up till now’. The second group of possible answers was: ‘never happened’; ‘happened during the COVID-19 pandemic, either only in selected phases or all through’; and ‘happened independently from the COVID-19 pandemic’ [22]. In line with what was performed with the other domains of QMNC, for the primary analysis of this domain, we combined, for each group of answers, the answers number two and three together since represented reports of lower or inappropriate quality of care, while for the secondary analysis, we presented the frequency of the answers number three (i.e. reports of inappropriate quality of care).

We performed a sensitivity analysis to assess the robustness of quality measure frequencies, including only respondents who answered all 40 quality measures and contributed to the total QMNC index, which aligns with other studies [47].

Both the total QMNC index and indexes by domains were calculated for the subsample of HWs, providing an answer to all 40 quality measures. We performed the Shapiro-Wilks test to evaluate the normal distribution of indexes. As indexes by domains were negatively skewed, the total QMNC index and indexes by domains were presented with median (MD) and interquartile range (IQR) overall and by country. Pairwise comparisons across the four domains overall and by country were tested with the Wilcoxon signed ranks test using Bonferroni adjustment.

We also investigated QMNC index trends over time during the COVID-19 pandemic and explored them in relation to the daily number of new COVID-19 cases. COVID-19 data were downloaded from the European Centre for Disease Prevention and Control database [48] for 11 countries, and for Switzerland, not included in the previous database, data was downloaded from the Swiss Federal Office of Public Health [49]. The total number of new daily COVID-19 cases in the 12 countries included in the present study was calculated. Moving averages spanning 14 previous days were presented for new daily COVID-19 cases and QMNC index. We assessed correlations among the two curves with the distance correlation coefficient (ρ), while the linear trend of the QMNC index over time was assessed through a linear regression using the moving average of the QMNC index as the independent variable.

Lastly, to explore differences in the total QMNC index among countries adjusted for self-described gender, professional qualification, facility type, working experience (years), and emergency phase of the COVID-19 pandemic in the European Union [50], we performed a multivariable linear regression with robust standard errors. Countries with <100 respondents were considered for this analysis as a single category. The categories with higher frequencies were selected as the reference category for each one of the previous variables. We used the Shapiro-Wilks test to assess the normal distribution of residuals of the model.

A two-tailed *P-*value <0.05 was considered statistically significant. Statistical analyses were performed using Stata/SE, version 14.0 (Stata Corporation, College Station, Texas, USA) and R, version 4.1.1 (R Core Team, Vienna, Austria).

### Ethics approval

The institutional review board of the Institute for Maternal and Child Health – IRCCS ‘Burlo Garofolo’ in Italy (IRB-BURLO protocol numbers 617/2016 and 05/2020) provided ethical clearance for this study. Further, ethical clearance was provided by ethical committees from Portugal (Instituto de Saúde Pública da Universidade do Porto – approval CE 20159, and Centro Hospitalar Universitário do Algarve – approval UAIF 101/2021), Norway (Norwegian Regional Committee for Medical Research Ethics – reference number 2020/213047), Germany (Bielefeld University Ethics Committee – reference number 2020-176) and Latvia (Rīgas Stradiņa Universitātes – approval 22-2/140/2021-16/03/2021). Other national ethical boards were approached for verification, and as no sensitive personal information was collected, no further formal ethical approval from other countries was required. We requested informed consent from all participants before answering the survey. The objectives and methods of the study were detailed, including rights regarding declining participation, and a privacy policy was made available online to responders before starting the survey. We ensured anonymity by not collecting any information that could disclose participants’ identities.

### Patient and public involvement

The team of the IMAgiNE EURO project includes more than 60 HWs involved in maternal or newborn care at different levels (midwives, nurses, OBGYN, paediatricians, physicians, lactation consultants, etc.). Patient advocates and representatives are also part of the IMAgiNE EURO team, contributing to the development of the study questionnaire, translation and validation, dissemination of survey links (through social media, HWs and professional associations), study design and data interpretation for this publication. A total of 600 HWs from six countries answered the survey during the questionnaire validation phase, contributing to optimising it [22].

## RESULTS

### Sample characteristics

**Figure 1:**
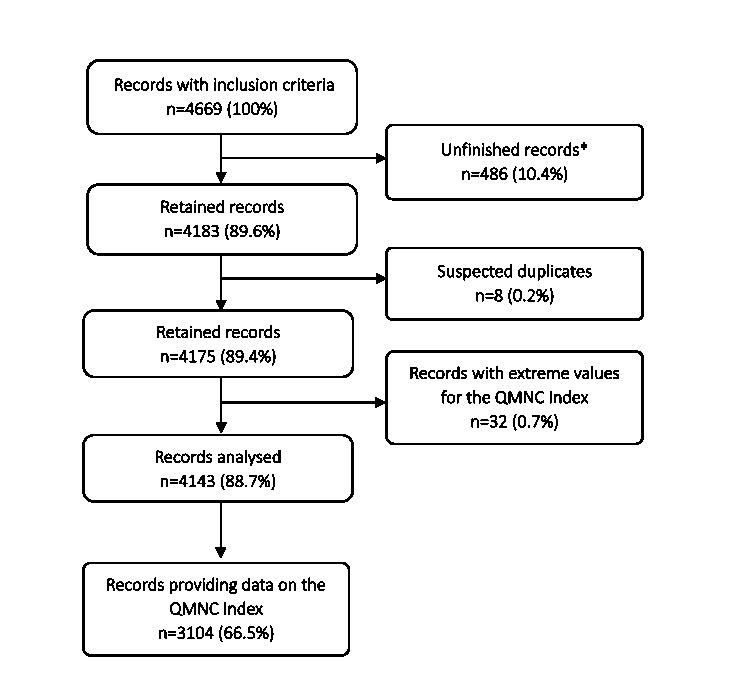
Flow diagram. Unfinished records are defined as records with missing data for 36 or more (≥90% of questions) quality measures (n = 40). QMNC – quality of maternal and newborn care.

Overall, 4669 HWs consented to participate and met the inclusion criteria. After the exclusion of unfinished records (n = 486, 10.4%), suspected duplicates (n = 8, 0.2%) and records with extreme values for the QMNC index (n = 32, 0.7%), we analysed 4143 records. Among these, 3104 (66.5%) were included in the QMNC index calculation (**Figure 1**). The number of participants varied among countries (**Table 1**), with 12 countries contributing with more than 100 HWs. Italy, Switzerland, and Romania contributed the largest samples (n = 589 (14.2%), n = 417 (10.1%), and n = 298 (7.2%), respectively) (Table S4 in the **Online Supplementary Document**).

**Table 1:** Characteristics of participants

Characteristics	Overall (n = 4143), n (%)
Country	
*Italy*	589 (14.2)
*Switzerland*	417 (10.1)
*Romania*	298 (7.2)
*Germany*	263 (6.3)
*Sweden*	219 (5.3)
*Greece*	217 (5.2)
*Portugal*	172 (4.2)
*Poland*	157 (3.8)
*Austria*	139 (3.4)
*Norway*	137 (3.3)
*Croatia*	134 (3.2)
*France*	115 (2.8)
Other*	272 (6.6)
*Missing†*	1014 (24.5)
Type of facility	
*Public*	3682 (88.9)
*Private*	461 (11.1)
Professional qualification	
*Midwife*	2500 (60.3)
*Nurse*	734 (17.7)
*Obstetrician gynaecologist*	468 (11.3)
*Neonatologist*	225 (5.4)
*Registrar/resident‡*	174 (4.2)
*General physician*	42 (1.0)
Gender (self-described)	
*Female*	2860 (69.0)
*Male*	204 (4.9)
*I prefer not to answer*	52 (1.3)
*Non-binary/gender fluid/agender/other*	13 (0.3)
*Missing*	1014 (24.5)
Working experience in years	
*<5*	679 (16.4)
*5–10*	609 (14.7)
*>10*	1843 (44.5)
*Missing†*	1012 (24.4)
Age in years	
*20–29*	505 (12.2)
*30–39*	943 (22.8)
*40–49*	835 (20.2)
*50–59*	656 (15.8)
*60–69*	185 (4.5)
*≥70*	6 (0.1)
*Missing†*	1013 (24.5)
Year of questionnaire completion	
*2021*	2185 (52.7)
*2022*	929 (22.4)
*2023*	2 (0.0)
*Missing†*	1027 (24.8)

*Other countries: Lithuania (n = 75, 2.4%), Slovenia (n = 64, 2.1%), Luxembourg (n = 48, 1.5%), Latvia (n = 27, 0.9%), Spain (n = 23, 0.7%), Bosnia and Herzegovina (n = 12, 0.4%), Ireland (n = 5, 0.2%), Montenegro (n = 4, 0.1%), Serbia (n = 4, 0.1%), UK (n = 4, 0.1%), Cyprus (n = 1, 0.0%), Denmark (n = 1, 0.0%), Macedonia (n = 1, 0.0%), Malta (n = 1, 0.0%), Tajikistan (n = 1, 0.0%), Ukraine (n = 1, 0.0%).

†Socio demographic questions were placed at the end of the questionnaire and more subjected to missing due to link abandonment.

‡A doctor in specialist training for obstetrics and gynaecology or neonatology.

Overall, 3682 HWs (88.9%) worked in public facilities, and 461 (11.1%) in private facilities (**Table 1**). Midwives and nurses were the most frequently reported type of health professionals (n = 2500 (60.3%) and n = 734 (17.7%), respectively). A high percentage of respondents were female (n = 2860, 69.0%); four out of 10 reported more than 10 years of work experience (n = 1843, 44.5%) and were 30–49 years old (n = 1778, 43.0%). About half of respondents completed the questionnaire in 2021 (n = 2185, 52.7%), and about a quarter in 2022 (n = 929, 22.4%).

### Quality measures

**Figure 2:**
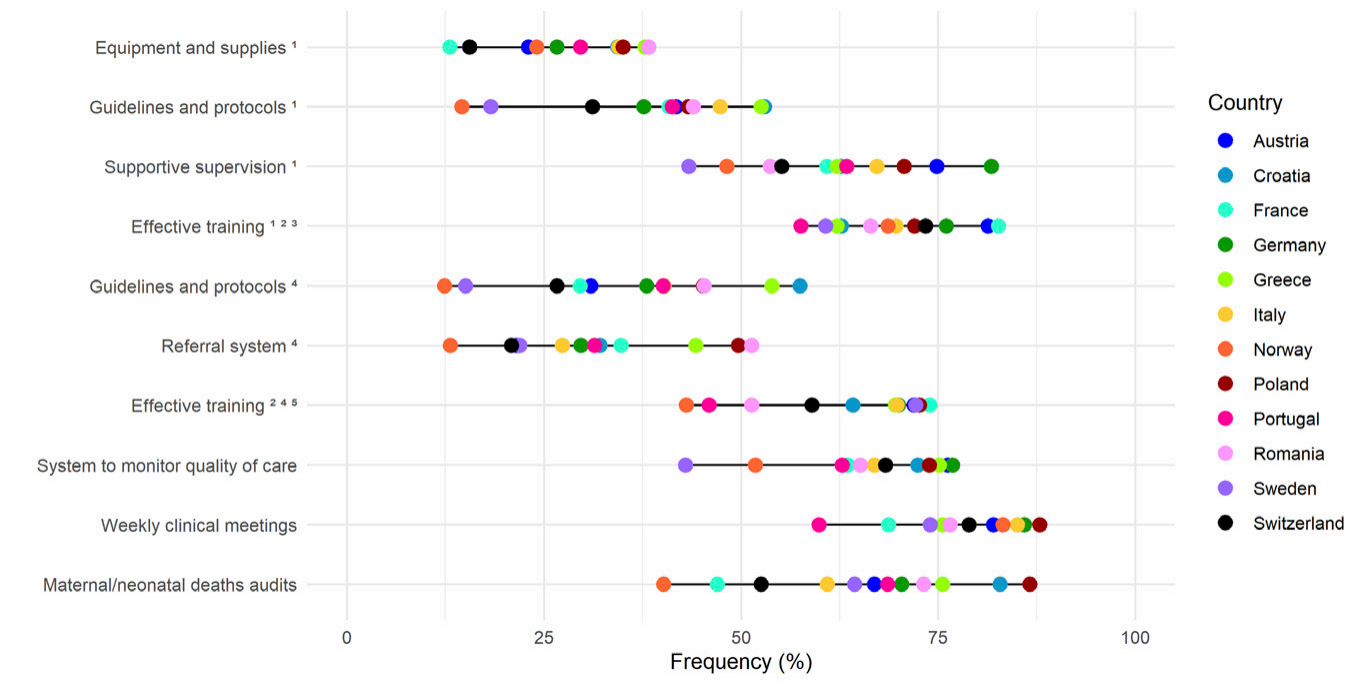
Need for improvement in the provision of care domain. Data are reported as country frequencies (coloured dots) and range of country frequencies (horizontal black lines). All quality measures in the domain of the provision of care are directly based on WHO standards. 1 – for case management of healthy women/newborns, 2 – at least one training event in the last three years, 3 – only for maternal area: partogram, foetal well-being, unnecessary caesarean section; only for neonatal area: breastfeeding promotion, skin-to-skin, standards precautions, 4 – for case management of emergencies, 5 – only for maternal area: postpartum haemorrhage, eclampsia, shoulder dystocia, pregnant woman cardiovascular arrest; only neonatal area: newborn resuscitation.

**Figure 4:**
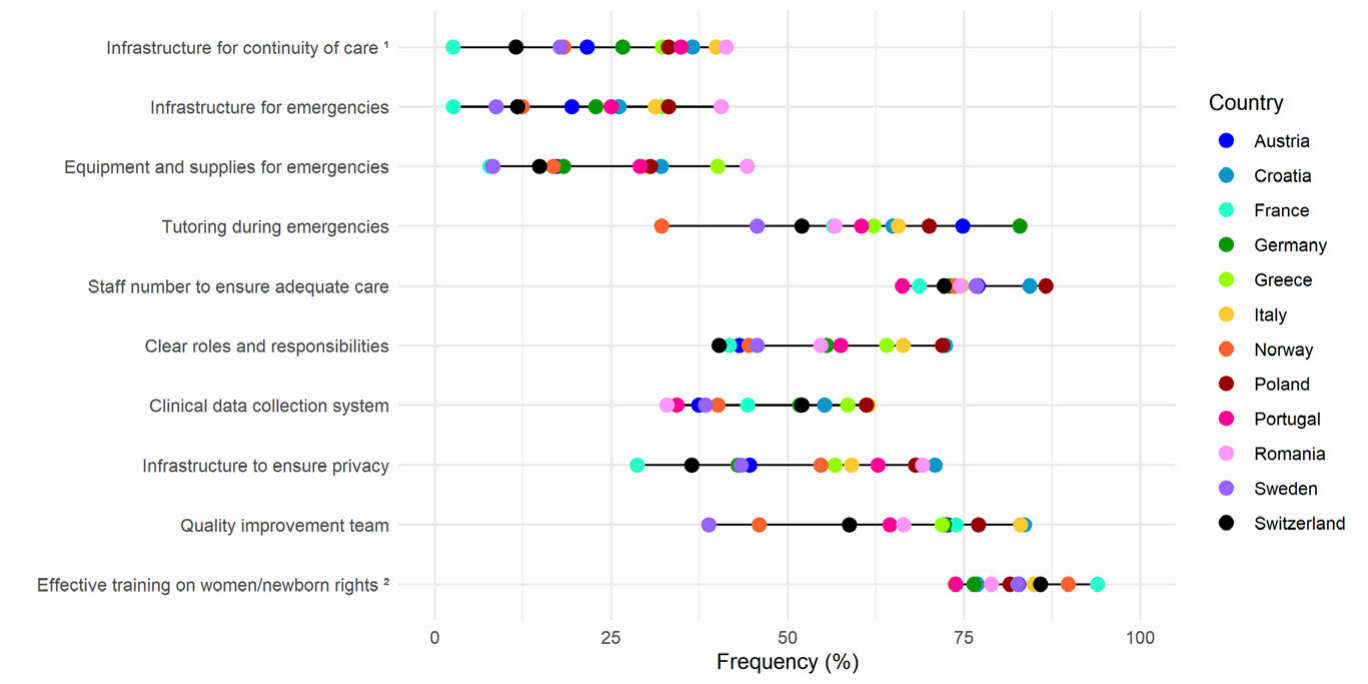
Need for improvement in the availability of motivated and competent human and physical resources domain. Data are reported as country frequencies (coloured dots) and range of country frequencies (horizontal black lines). All quality measures in the domain of the availability of motivated and competent human and physical resources are directly based on WHO standards. 1 – for healthy women/newborns care, 2 – at least one training event in the last three years.

When calculated on the overall sample, all quality measures suggested gaps in QMNC, as perceived by HWs. The frequency of ‘need for improvement’ (primary analysis) was overall >20% for 39 out of 40 quality measures (**Figure 2**, **Figure 4**, Tables S5–8 in the **Online Supplementary Document**). The frequency of ‘need of significant improvement’ (secondary analysis) was higher than 20% for 13 of the 40 quality measures. These 13 quality measures were distributed in all domains except the COVID-19 domain. Four quality measures pertained to the domain of provision of care, five to the experience of care, and four to the domain of resources (Tables S9–12 in the **Online Supplementary Document**). Additionally, large variations in frequencies across countries were observed for all 40 quality measures.

Regarding the domain of provision of care (**Figure 2**), the quality measures with the highest reported ‘need for improvement’ frequencies were effective training on case management of healthy women/newborns (overall 66.3%; ranging from 57.6% in Portugal to 82.6% in France) and weekly clinical meetings to discuss relevant cases (overall 63.8%; ranging from 59.9% in Portugal to 87.9% in Poland). More than half (56.6%) of HWs highlighted the ‘need for improvement’ in systems to routinely monitor QMNC (ranging from 42.9% in Sweden to 76.8% in Germany). In the secondary analysis, i.e. calculating only the frequency of answers ‘it needs significant improvement,’ training on management of emergencies presented the highest frequency (overall 44.1%; ranging from 80.3% in Poland to 38.3% in France) (Tables S5–9 in the **Online Supplementary Document**).

**Figure 3:**
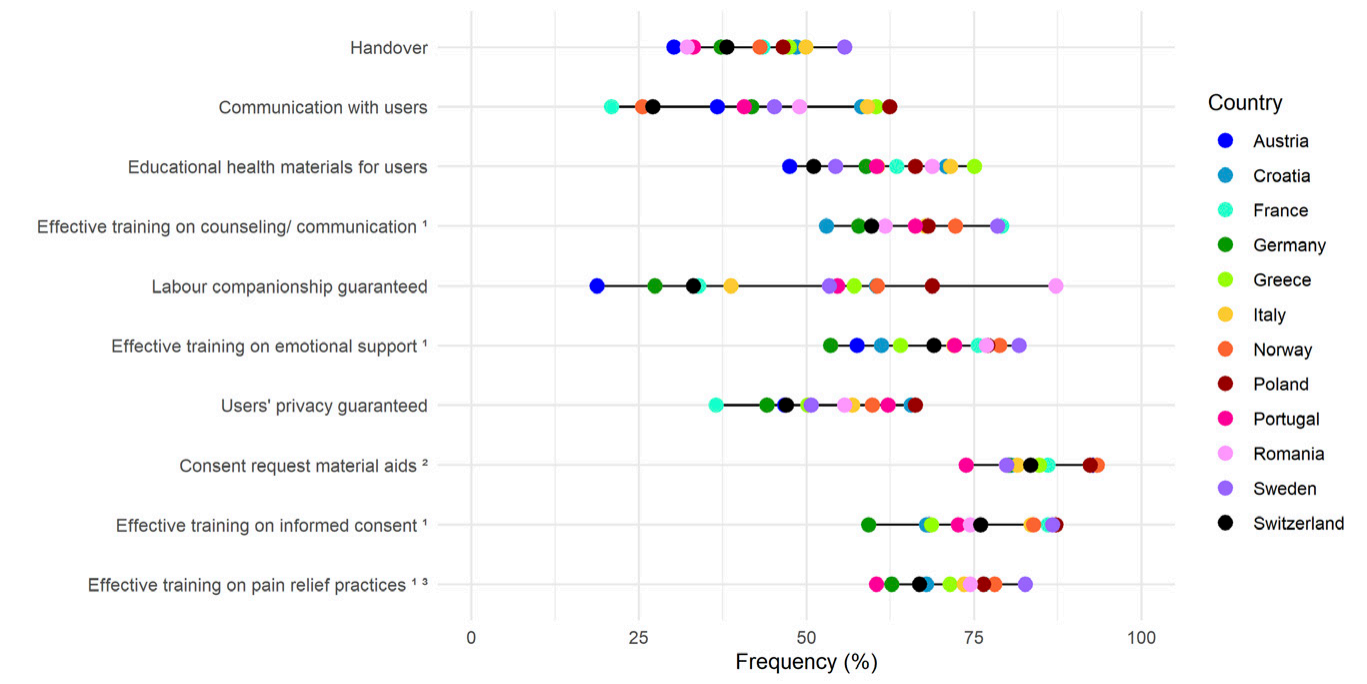
Need for improvement in the experience of care domain. Data are reported as country frequencies (coloured dots) and range of country frequencies (horizontal black lines). All quality measures in the domain of experience of care are directly based on WHO standards. 1 – at least one training event in the last three years; 2 – only for maternal area: regular orientation sections for women during pregnancy, written/digital material for consent before ceasarean section, induction of labour; only for neonatal area: regular orientation sections for women during pregnancy, written/digital material for consent before newborn vitamin K administration, newborn eye drops/ointment application; 3 – only for maternal area: pharmacological and non-pharmacological pain relief on labour; only for neonatal area: prevention/management of newborn’s pain.

In the domain of experience of care (**Figure 3**), the quality measures with the highest overall ‘need for improvement’ frequencies were the availability of informed consent job aids such as, but not limited to, written or digital materials (66.9%; ranging from 73.8% in Portugal to 93.4% in Norway) and training events on how to appropriately offer informed choices (61.7%; ranging from 59.3% in Germany to 87.3% in Poland). The quality measure with the highest variation across countries was the possibility for women to have a labour companion of choice, ranging from 18.7% in Austria to 87.2% in Romania. In the secondary analysis, 42.7% of respondents (ranging from 71.3% in Poland to 33.2% in Germany) reported the ‘need of significant improvement’ on training on informed consent (Tables S6–10 in the **Online Supplementary Document**).

Regarding the domain of availability of motivated and competent human and physical resources (**Figure 4**, Table S7 in the **Online Supplementary Document**), the quality measures with the highest overall ‘need for improvement’ frequencies were training events covering the rights of women and newborns (overall 65.5%; ranging from 73.7% in Greece to 93.9% in France) and availability of HWs, i.e. staff number, to ensure adequate care (overall 64.8%; ranging from 66.3% in Portugal to 86.6% in Poland). More than half of the total sample (54.9%) reported the need for a dedicated team/unit for QMNC improvement (ranging from 38.8% in Sweden to 83.6% in Croatia). In the secondary analysis, in line with the primary analysis, the training on the rights of women and newborns was the quality measure with the highest frequency (overall 49.8%; ranging from 82.6% in France to 48.4% in Greece) (Table S11 in the **Online Supplementary Document**).

**Figure 5:**
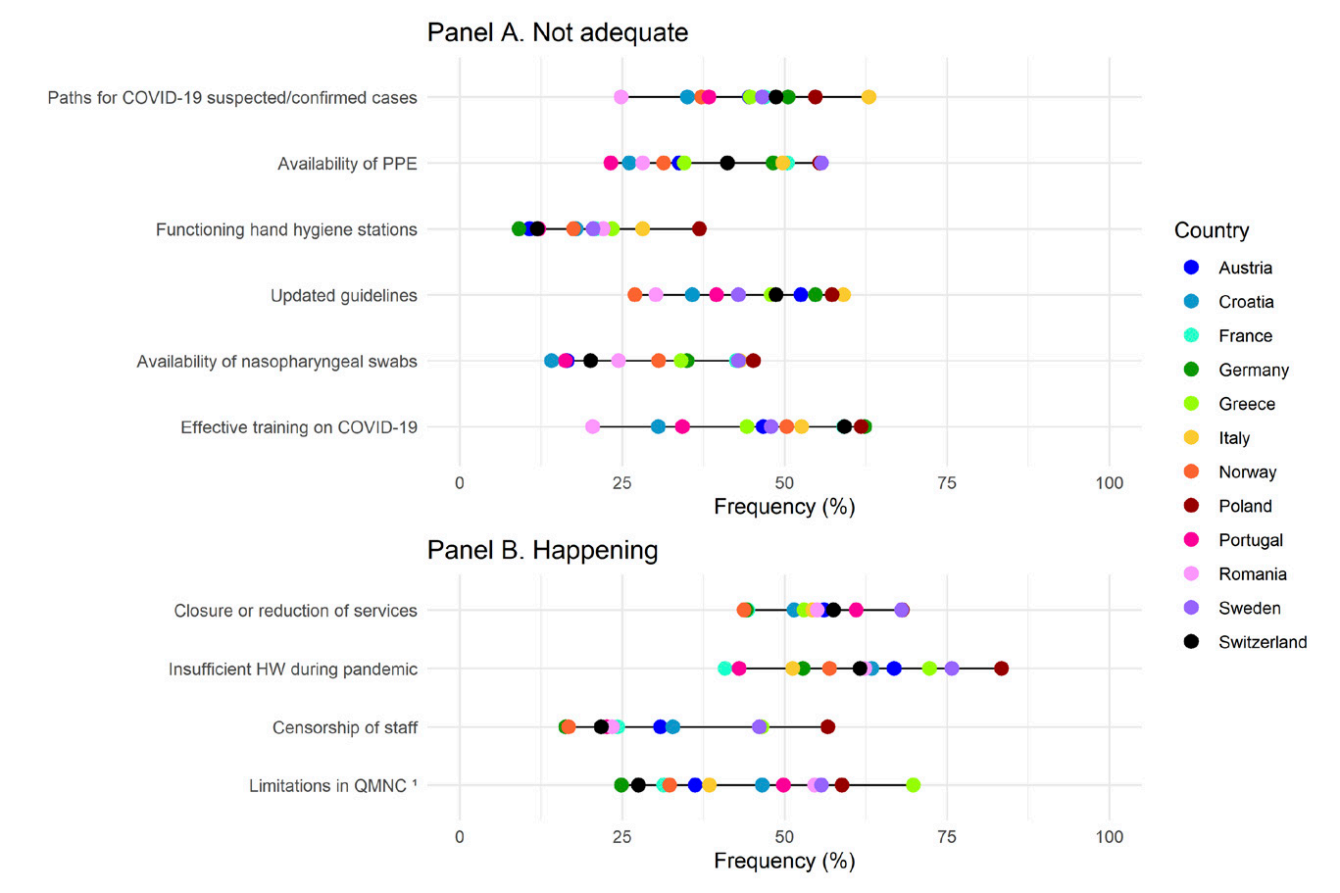
Findings in the COVID-19 domain. **Panel A.** ‘Not always existing and/or not fully adequate’ OR ‘Never existed and/or never adequate since the beginning of pandemic up till now.’ **Panel B.** ‘Happened during the COVID-19 pandemic’ OR ‘Happened independently from the COVID-19 pandemic.’ Data are reported as country frequencies (coloured dots) and range of country frequencies (horizontal black lines). 1 – Frequency is calculated on seven indicators contributing to the same quality measure: increased medicalisation and/or limitations on companionship, restrictions on movements during labour, limitations on pain relief procedures, limitations on rooming-in practices without clinical indications, limitations on breastfeeding without clinical indications, limitations on skin to skin in the absence of clear medical indications. HW – health workers, PPE – personal protective equipment; QMNC – quality of maternal and newborn care.

Regarding organisational changes due to the COVID-19 pandemic (**Figure 5**, Table S8 in the **Online Supplementary Document**), quality measures with the highest overall frequencies of ‘not adequate’ were insufficient HWs numbers during the pandemic (overasll 46.9%; ranging from 40.9% in France to 83.4% in Poland) and closure of wards or routine services reduction (overall 43.7%; ranging from 44.1% in German to 68.2% in Poland). The ‘not adequate’ lowest frequency was found for the presence of functioning and accessible hand hygiene stations (overall 15.7%; ranging from 9.1% in Germany to 36.9% in Poland). Around one-third (32.0%) of HWs reported that personal protective equipment distribution was inadequate (ranging from 23.3% in Portugal to 55.7% in Sweden). In the primary analysis, 22.4% of staff reported censorship (silencing of staff) to avoid reporting inadequate procedures (ranging from 16.3% in Germany to 56.7% in Poland). These frequencies were lower in the secondary analysis (overall 10.6%; ranging from 5.8% in Norway to 34.7% in Sweden), with this quality measure presenting the second highest frequency in this domain (Table S12 in the **Online Supplementary Document**).

### Sensitivity analyses

The findings of the sensitivity analyses were substantially consistent with the findings of the primary analysis (Tables S13–17 in the **Online Supplementary Document**), with respondents reporting higher frequencies of ‘need for improvement’ than in the primary analysis for 37 out of 40 quality measures. Frequencies from sensitivity analyses resulted lower than in the primary analysis for only three quality measures – the availability of guidelines/protocols (39.3% vs 40.2%), availability of equipment/supplies for case management of healthy women/newborns (27.7% vs 29.2%), and infrastructure for continuity of care for healthy women/newborns (27.6% vs 29.6%).

### QMNC index

The total QMNC index (possible score ranging 0–400) had overall low values (MD = 260.24; IQR = 208.24, 308.93) and varied substantially between countries (*P* < 0.001) (e.g. in Poland MD = 210.60 (IQR = 155.71, 273.57) and Norway MD = 277.86 (IQR = 244.32, 308.30)) (Table S18 in the **Online Supplementary Document**).

**Figure 6:**
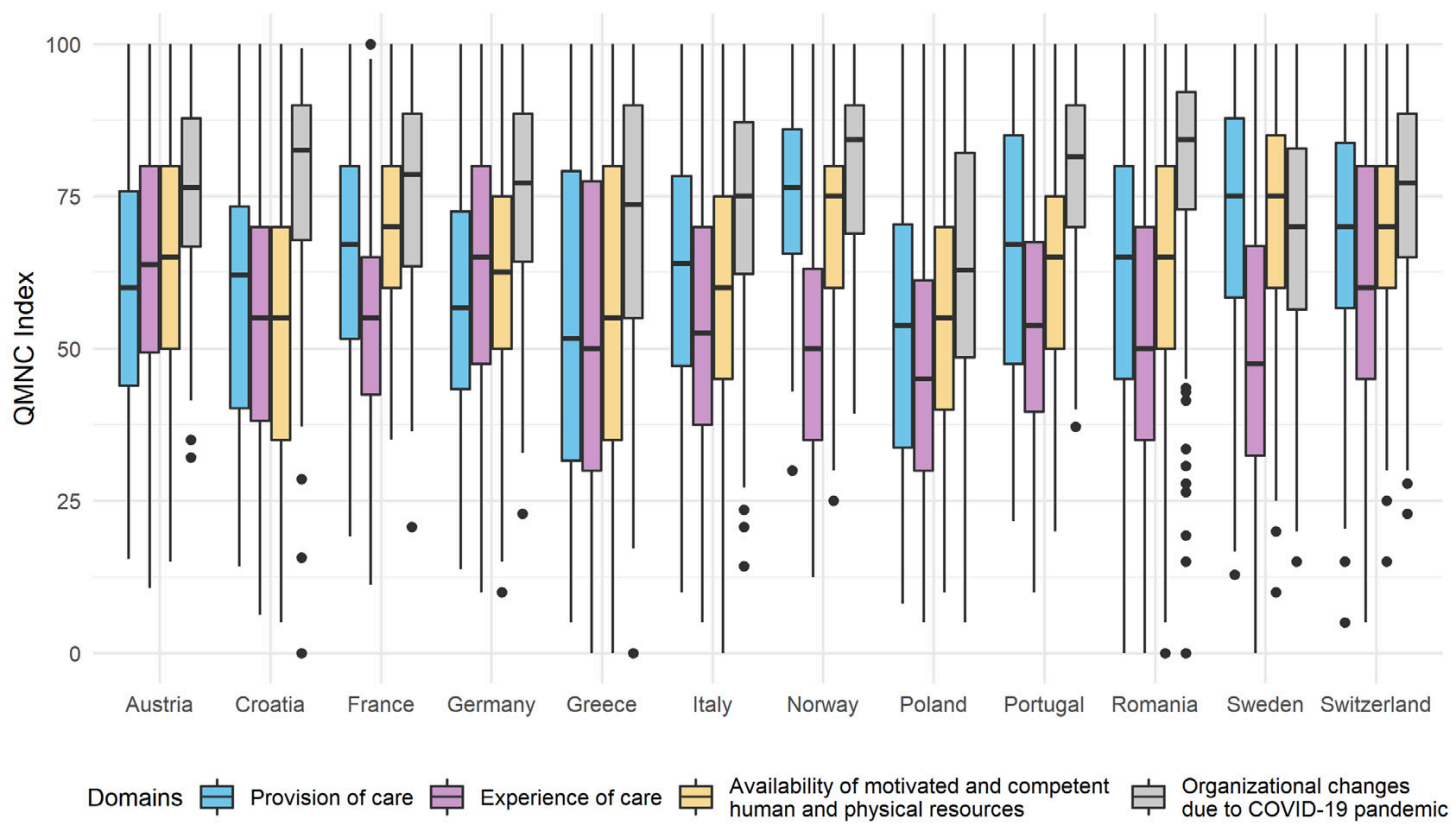
QMNC Indexes by country and by domain. Data are reported using box plots (showing median and interquartile range), whiskers (representing 1.5 times the interquartile range) and dots (the extreme values) (n = 3093). QMNC – quality of maternal and newborn care.

The domain of experience of care (MD = 55.00, IQR = 40.00, 72.50) was rated with the lowest scores (Table S19 in the **Online Supplementary Document**). When data were analysed by country, the scores on the experience of care domain were significantly lower than scores of other domains in eight out of 12 countries (i.e. France, Italy, Norway, Poland, Portugal, Romania, Sweden, and Switzerland) (**Figure 6**, Tables S18–19 in the **Online Supplementary Document**).

**Figure 7:**
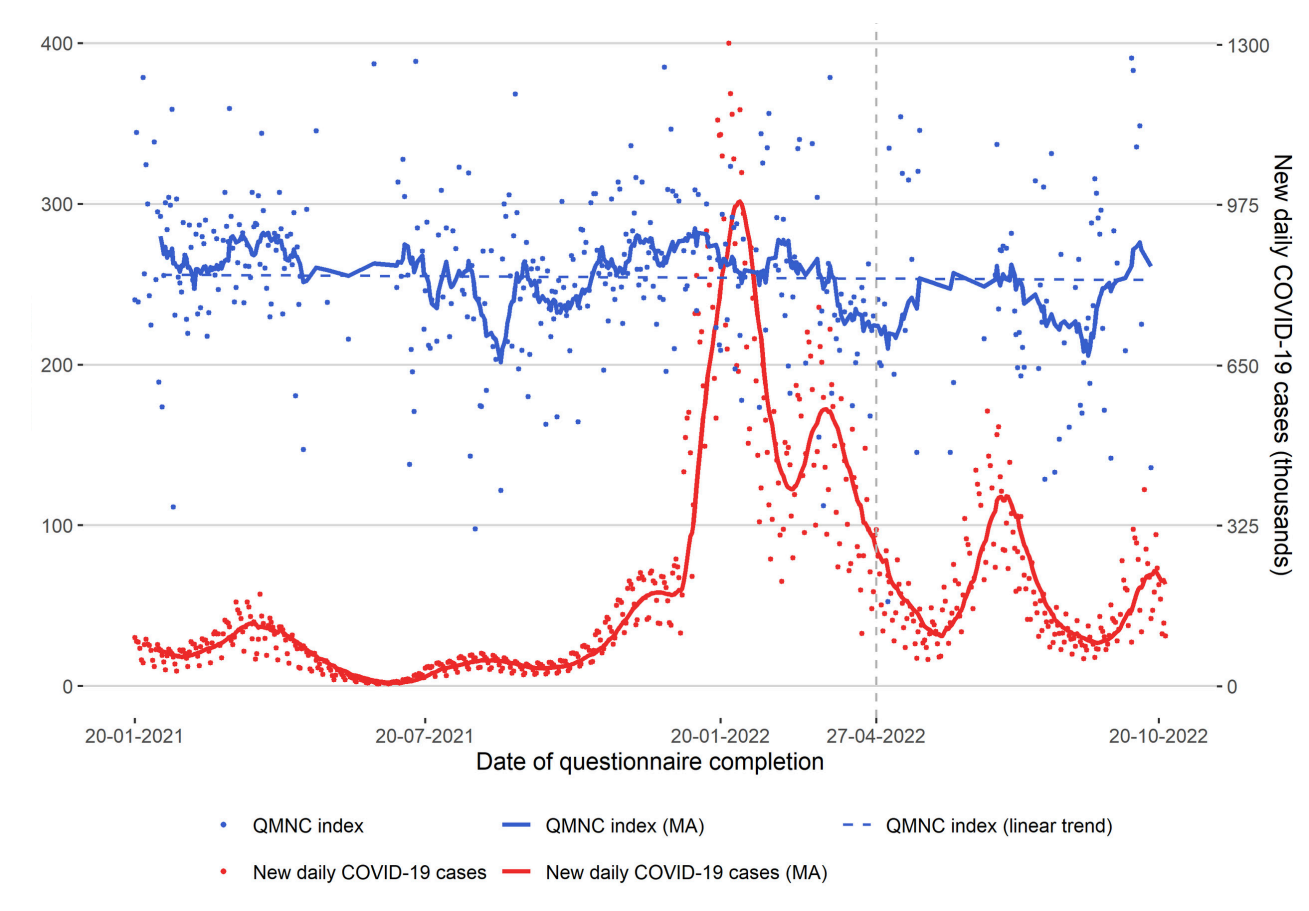
Total QMNC index over time. The vertical grey dashed line shows the date of 27 April 2022, when was declared that the European Union was moving out of the emergency phase of the COVID-19 pandemic (Communication from the Commission to the European Parliament, the Council, the European Economic and Social Committee and the Committee of the Regions, COVID-19 - Sustaining EU Preparedness and Response: Looking ahead) [50]. MA – moving average, QMNC – quality of maternal and newborn care.

When trends in the total QMNC index were analysed over time (**Figure 7**), moving average values tended to slightly move up and down (in between the scores 204.5–283.1), with a slight and significant monthly linear decrease (β = –1.06; 95% confidence interval (CI) = –1.56, –0.57, *P* < 0.001). There was no significant correlation between moving averages of the total QMNC index and new COVID-19 cases (ρ = 0.17; *P* > 0.05).

### Multivariate analyses

When adjusted for other variables, compared to the reference country (Italy), HWs from Poland reported significantly lower QMNC index MD scores (β = –23.68, *P* < 0.001). Norway (β = 41.81, *P* < 0.001), Austria (β = 34.23, *P* < 0.001), Switzerland (β = 33.23, *P* < 0.001), Sweden (β = 17.73, *P* = 0.001), Germany (β = 17.56, *P* < 0.001), and France (β = 11.83, *P* = 0.045) reported significantly higher scores. All professional qualifications compared to midwives, male HWs and HWs working in private facilities evaluated the QMNC with significantly higher index scores. Less experienced HWs, i.e. those with less than 10 years of experience, evaluated the QMNC with significantly lower values. There was no significant change in the QMNC index after the date when it was declared that European Union was moving out of the emergency phase of the COVID-19 pandemic (Table S20 in the **Online Supplementary Document**).

## DISCUSSION

This is the first multicountry study documenting HWs perspectives on the QMNC around the time of childbirth at health facilities in the WHO European Region countries, using the WHO standards [15] as a reference. Overall, although good practices were also described, QMNC gaps were reported in all 12 countries without a significant association with COVID-19 pandemic trends. Many areas of maternal and newborn care need improvements, according to HWs, particularly those related to the effectiveness of training, informed consent practices, and availability of HWs. Sensitivity and multivariate analyses confirmed the results of the primary descriptive analysis, with large variations of QMNC by country.

The findings of this study align with the few existing quantitative studies reporting on HWs’ perspectives on QMNC in high-income countries [17,32,35,51]. A WHO report ‘Health and Care Workforce in Europe: Time to Act’ underscores that all countries in the European Region face severe problems related to shortages of health professionals, inefficient work organisation, and insufficient investment in the development of the health workforce, leading to suboptimal provision of health and care services [52]. According to other WHO reports [19,53–55], pre-existing inequities added to the impact of the COVID-19 pandemic on health systems might have, to varying extents, delayed progress towards the health-related Sustainable Development Goals by 2030, irrespective of countries’ income level.

In addition to the WHO reports [52–55], several systematic reviews highlighted an insufficient number of HWs to enable the provision of quality care, affecting health systems before and during the pandemic, with considerable inequities across countries worldwide [56–60]. Workforce ageing in the last decade, increased absenteeism, increased number of HWs deaths during the COVID-19 pandemic and increased migration of health care workers from specific countries are listed among key causes for the lack of sufficient health care workforces in the WHO Europe Region [61].

With respect to another key finding of this study – the need to provide effective training to HWs – the WHO ‘Working for Health 2022–30 Action Plan: Education and Employment’ highlights that adequate investment in HWs education, including post-service professional, technical, and vocational education and training, is crucial for enhancing working conditions and for attracting and retaining professionals in the health and care sector [62]. It is difficult to quantify the full negative impact of the lack of effective HWs training on key areas of QMNC, such as providing emotional support, universal rights of childbearing women, and communication on the QMNC delivered and health outcomes. Besides effective training programs, concrete resources (e.g. personal, financial, etc.) are also necessary to improve QMNC, and plausibly, combining both of these factors may be one of the core factors constraining the achievement of high QMNC. Solutions need to be tailored to each setting based on local priorities and sustainability. Future implementation research shall further explore how to better use the findings from this study across different settings and which could be the most effective strategies for delivering those specific training packages and monitoring learning outcomes.

Within a health care context, the high censorship rates (silencing of staff) to avoid reporting inadequate procedures described by respondents across countries is extremely concerning for patient safety. Recent systematic reviews have associated the silencing of staff with a facility culture which discourages transparency, authoritarian organisational hierarchy, inappropriate supervisor behaviour, discrimination issues, neglected care, and lack of resources [63–66]. Future research from IMAgiNE EURO, including analyses of qualitative data, will help to clarify the complex nature of these findings in each country, and future rounds of data collection will help to clarify if this phenomenon and other findings of this study will change after the end of the emergency phase of the COVID-19 pandemic. Further studies should explore how the institutional culture may have affected the willingness to participate in an anonymous survey on quality of care and how national, institutional and individual culture may have affected participants’ responses.

Not surprisingly, our data show large variation across countries on HWs reports about the lack of a routine system for monitoring QMNC and lack of a dedicated team for improving it. Many approaches to quality improvement stress the importance of local ownership and leadership [67,68]. Identifying and involving multidisciplinary teams who will be responsible for the implementation of quality improvement initiatives at the facility level from the beginning of the process is crucial. This strategy can foster HWs’ motivation to change and promote positive attitudes and behaviours that ultimately improve women’s experiences and health outcomes around childbirth [69–71].

When compared to the assessment of QMNC from the maternal perspective, gaps reported by HWs can directly negatively affect women’s experiences during hospitalisation for childbirth. For instance, but not limited to, in Lazzerini et al. [23], 11 198 women (62.0%) from 12 WHO European countries highlighted that a companion of choice was not allowed during childbirth, and 18.2% (n = 3287) of women who underwent labour complained about lack of privacy. In our study, 39.9% of HWs perceived the need to improve companionship during hospitalisation for childbirth, and 43.4% confirmed that facility infrastructure is insufficient to ensure service users’ privacy. This comparison suggests that some practices or limitations on practices (such as guaranteeing companionship) are more visible or important to women, whereas others (such as infrastructural challenges) may be more visible or important to HWs. On the other hand, when considering other key indicators during the COVID-19 pandemic, a higher percentage of Swedish women (62.5%) reported that HWs were not always using personal protective equipment [24], and the information was confirmed for more than half of our HWs sample (55.7%) in Sweden. We acknowledge that samples and study periods of the IMAgiNE EURO maternal surveys [23,24] vs our study using HWs survey [22] are not directly comparable. However, the results of the two surveys complement each other and provide a more comprehensive picture of QMNC delivered in the region, suggesting that multiple data sources are valuable for health policy development and for continuous monitoring. Further analysis comparing results obtained from mothers’ and HWs’ perspectives will be provided in future IMAgiNE EURO project publications, particularly for the experience of care quality measures.

Careful comparison between countries is warranted due to potential variations in data collection time points. This online survey may have been affected by self-selection and social desirability bias. The self-administration mode of the questionnaire, the anonymity of online responses (i.e. no information was collected that might have identified the health worker, his/her computer internet protocol address or the specific institution), the preface of questions explaining areas to be assessed [22], and the exclusion of questionnaires with the same pattern of answers for all quality measures might have contrasted or avoided this type of bias to some extent [72–74]. Moreover, we can’t predict in which direction this may have affected the study findings, either in an overestimation of the QMNC or an underestimation. However, the lack of official comparable data on the number of health workforce in most European countries [52] limits any conclusion regarding the representativeness of our sample or the generalisability of results. Multivariate analyses corrected findings on QMNC index for professional qualification and other characteristics of responders. Facilities interested in knowing results specifically related to their setting may replicate the IMAgiNE EURO survey (currently available in 17 languages) among the facility’s HWs. The higher response rate from midwives suggests that, besides being the most frequent provider of maternal and newborn care in many countries, as a group, when invited, they may be more willing to provide their perspectives for QMNC improvement compared to other categories of HWs. Strategies for enhancing participation from doctors and other categories of HWs may be used to optimise the representativeness of the sample in each country/facility.

We acknowledge that HWs’ perceptions of QMNC may be affected by national/institutional/individual culture and social or individual expectations [71,75–77]. However, most of the quality measures explored were objective and relatively easy to recall (e.g. ‘there is a sufficient number of health care professionals,’ ‘there is a clinical data collection system,’ etc.), which may have increased reliability and comparability of our data. Other upcoming publications of the IMAgiNE EURO project will focus on analysing and comparing detailed results by country and diverse geographic areas, and exploring indicators trends over time.

The variables placed at the end of the questionnaire had a high number of missing data. In designing questionnaires, there is often a tension between the desire to collect comprehensive data and acceptability for respondents, and it is not uncommon to have a considerable amount of missing data from online surveys [78–81]. The sensitivity analysis included only respondents who answered all key quality measures and demonstrated the robustness of the findings. Future versions of the IMAgiNE EURO HWs questionnaire may consider reducing the number of questions.

Meanwhile, in addition to previous data [16,19,52–55,62], this study calls for concrete actions. Gaps reported can negatively affect both women’s childbirth experiences, health outcomes, and HWs motivation and/or performance. They underline the need to implement quality improvement initiatives at all health system levels. Additionally, study findings call for further monitoring of QMNC, as recommended by WHO [15,52]. The IMAgiNE EURO project provides data overall collected through a systematic methodology – two validated questionnaires [21,22] on 80 WHO standard-based quality measures (40 related to service providers and 40 related to service users) – and this is a concrete opportunity for data monitoring over time and across settings and integration into official statistics. IMAgiNE EURO data, providing two complementary perspectives, can also be triangulated with other data sources and can support a constructive dialogue between HWs, other stakeholders and decision-makers to prioritise areas for improvement and set specific initiatives. These data, together with findings from existing reports [19,52–55,62], should be proactively used for planning QMNC improvements in the region. Data collected can be used as evidence to design improvement initiatives tailored to each setting, aiming to promote a positive working environment for HWs and improve childbirth experiences for women and families and health outcomes.

## CONCLUSIONS

In this study, HWs from 12 WHO European countries reported QMNC gaps and inequities across countries during facility childbirth using WHO standards as a reference. Findings strongly suggest the need for more investments in improving and actively monitoring QMNC over time and across different settings using HWs’ perspectives. Key areas for concrete actions include ensuring that adequate skilled HWs are available for all women and newborns, HWs have access to effective training programs with learning outcomes monitoring and appropriate job aids for consent requests, and correcting concerning practices such as silencing staff.

## Additional material


Online Supplementary Document
