# Supplementary material for: Health workers’ perspectives on the quality of maternal and newborn health care around the time of childbirth: Results of the Improving MAternal Newborn carE in the EURO Region (IMAgiNE EURO) project in 12 countries of the World Health Organization European Region
Source: J Glob Health. 2024 Sep 6;14:04164. doi: 10.7189/jogh.14.04164 (PMC11377968; doi:10.7189/jogh.14.04164)
Supplement: Online Supplementary Document [file jogh-14-04164-s001.pdf]

Health workers' perspectives on the quality of facility-based maternal and newborn care around childbirth in WHO European region: results of IMAgiNE EURO survey in 12 countries

Supplementary files

Table of Contents

Supplementary Table 1. The Strengthening the Reporting of Observational Studies (STROBE) Checklist 2

Supplementary Figure 1. Summary steps of questionnaire development and validation 4

Supplementary Table 2. Data collection period by language 5

Supplementary Table 3. Quality of maternal and neonatal care index score system 6

Supplementary Table 4. Sample characteristics by country 7

Supplementary Table 5. Need for improvement in provision of care domain 9

Supplementary Table 6. Need for improvement in experience of care domain 10

Supplementary Table 7. Need for improvement in availability of motivated and competent human and physical resources domain 11

Supplementary Table 8. Frequency of not adequate or happened during the COVID-19 pandemic in the organizational changes due to COVID-19 pandemic domain 12

Supplementary Table 9. Provision of care domain secondary analysis: need for significant improvement 13

Supplementary Table 10. Experience of care domain secondary analysis: need for significant improvement 14

Supplementary Table 11. Availability of motivated and competent human and physical resources domain secondary analysis: need for significant improvement 15

Supplementary Table 12. Organizational changes due to COVID-19 pandemic domain secondary analysis: never adequate independently from pandemic 16

Supplementary Table 13. Sensitivity analysis - characteristics of respondent 17

Supplementary Table 14. Sensitivity analysis: Provision of care domain - frequency of need for improvement 18

Supplementary Table 15. Sensitivity analysis: Experience of care domain - frequency of need for improvement 19

Supplementary Table 16. Sensitivity analysis: Availability of motivated and competent human and physical resources domain - frequency of need for improvement 20

Supplementary Table 17. Sensitivity analysis: Organizational changes due to COVID-19 pandemic domain - frequency of not adequate or happened during the COVID-19 pandemic 21

Supplementary Table 18. QMNC Index by country and by domain 22

Supplementary Table 19. Pairwise comparisons across four domains, overall and by country 23

Supplementary Table 20. Multivariate linear regression model with QMNC Index as outcome 24

Supplementary Table 1. The Strengthening the Reporting of Observational Studies (STROBE) Checklist

	Item No	Recommendation	Pages
**Title and abstract**	1	(*a*) Indicate the study's design with a commonly used term in the title or the abstract	4
		(*b*) Provide in the abstract an informative and balanced summary of what was done and what was found	4
**Introduction**			
Background/rationale	2	Explain the scientific background and rationale for the investigation being reported	6
Objectives	3	State specific objectives, including any prespecified hypotheses	6-7
**Methods**			
Study design	4	Present key elements of study design early in the paper	7
Setting	5	Describe the setting, locations, and relevant dates, including periods of recruitment, exposure, follow-up, and data collection	7-8
Participants	6	(*a*) Give the eligibility criteria, and the sources and methods of selection of participants	7-8
Variables	7	Clearly define all outcomes, exposures, predictors, potential confounders, and effect modifiers. Give diagnostic criteria, if applicable	9-10
Data sources/ measurement	8*	For each variable of interest, give sources of data and details of methods of assessment (measurement). Describe comparability of assessment methods if there is more than one group	5-6
Bias	9	Describe any efforts to address potential sources of bias	7-11
Study size	10	Explain how the study size was arrived at	9
Quantitative variables	11	Explain how quantitative variables were handled in the analyses. If applicable, describe which groupings were chosen and why	9-11
Statistical methods	12	(*a*) Describe all statistical methods, including those used to control for confounding	9-11
		(*b*) Describe any methods used to examine subgroups and interactions	9-11
		(*c*) Explain how missing data were addressed	9-11
		(*d*) If applicable, describe analytical methods taking account of sampling strategy	9-11
		(*e*) Describe any sensitivity analyses	10
**Results**			

Participants	13*	(a) Report numbers of individuals at each stage of study—eg numbers potentially eligible, examined for eligibility, confirmed eligible, included in the study, completing follow-up, and analysed	12
		(b) Give reasons for non-participation at each stage	12
		(c) Consider use of a flow diagram	figure 1
Descriptive data	14*	(a) Give characteristics of study participants (eg demographic, clinical, social) and information on exposures and potential confounders	13-14
		(b) Indicate number of participants with missing data for each variable of interest	13-14
Outcome data	15*	Report numbers of outcome events or summary measures	14-15
Main results	16	(*a*) Give unadjusted estimates and, if applicable, confounder-adjusted estimates and their precision (eg, 95% confidence interval). Make clear which confounders were adjusted for and why they were included	13-16
		(*b*) Report category boundaries when continuous variables were categorized	-
		(*c*) If relevant, consider translating estimates of relative risk into absolute risk for a meaningful time period	-
Other analyses	17	Report other analyses done—eg analyses of subgroups and interactions, and sensitivity analyses	15-16
**Discussion**			
Key results	18	Summarise key results with reference to study objectives	16-17
Limitations	19	Discuss limitations of the study, taking into account sources of potential bias or imprecision. Discuss both direction and magnitude of any potential bias	19-20
Interpretation	20	Give a cautious overall interpretation of results considering objectives, limitations, multiplicity of analyses, results from similar studies, and other relevant evidence	17-20
Generalisability	21	Discuss the generalisability (external validity) of the study results	17-20
**Other information**			
Funding	22	Give the source of funding and the role of the funders for the present study and, if applicable, for the original study on which the present article is based	26

Notes: * Give information separately for exposed and unexposed groups.

Supplementary Figure 1. Summary steps of questionnaire development and validation

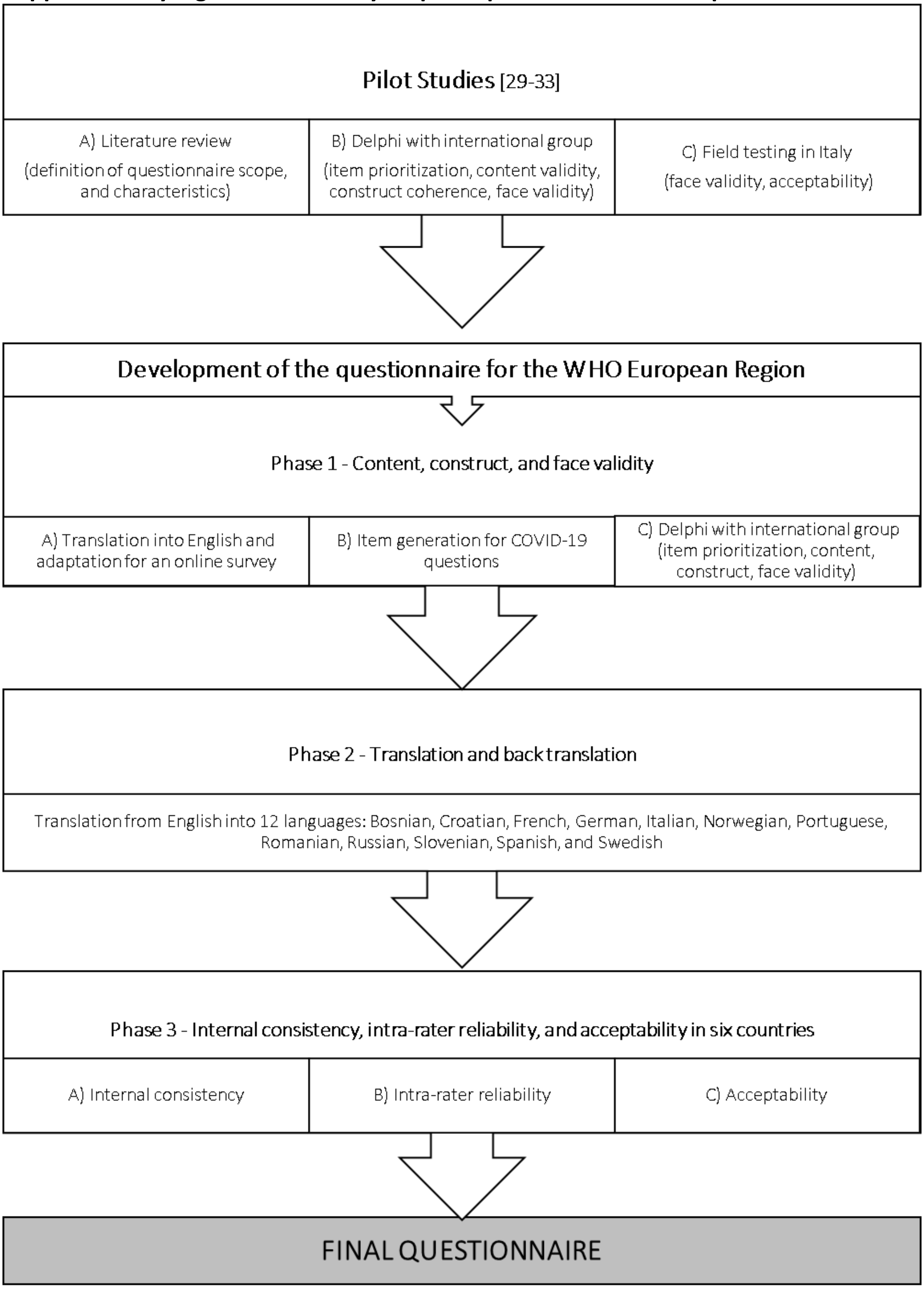

Note: Valente EP, Covi B, Mariani I, et al. WHO Standards-based questionnaire to measure health workers' perspective on the quality of care around the time of childbirth in the WHO European region: development and mixed-methods validation in six countries. BMJ Open 2022;12:e056753. doi:10.1136/bmjopen-2021-056753

Supplementary Table 2. Data collection period by language

	Languages*	Date of online availability	Country team responsible
**Dissemination of link under direct responsibility of a specific country team**	German	05/02/2021	Austria, Germany, Luxembourg, and Switzerland
	French	13/02/2021	France, Luxembourg, and Switzerland
	Slovenian	15/02/2021	Slovenia
	Spanish	08/03/2021	Spain
	Italian	02/07/2021	Italy and Switzerland
	Romanian	07/07/2021	Romania
	Portuguese	19/07/2021	Portugal
	Bosnian	19/07/2021	Bosnia and Herzegovina
	Norwegian	12/08/2021	Norway
	Croatian	16/08/2021	Croatia
	Polish	22/08/2021	Poland
	Swedish	29/09/2021	Sweden
	Lithuanian	27/01/2022	Lithuania
	Latvian	01/02/2022	Latvia
	Greece	22/07/2022	Greece
**Dissemination of link not under direct responsibility of a specific country team**	English	05/02/2021	NA
	Russian	30/11/2021	

Notes: *All links are always available to all countries after the start date.
Abbreviation: NA = not applicable.

Supplementary Table 3. Quality of maternal and neonatal care index score system

Domains of health worker's questionnaire	Number of questions	Score options for each answer	Total score (range)
Provision of care	10	0 to 10	0 to 100
Experience of care	10	0 to 10	0 to 100
Availability of physical and human resources	10	0 to 10	0 to 100
Organizational changes due to COVID-19 pandemic	10	0 to 10	0 to 100
**TOTAL QMNC Index – the sum of four score domains**	**40**	**0 to 10**	**0 to 400**

Abbreviation: QMNC = Quality of maternal and neonatal care

Supplementary Table 4. Sample characteristics by country

	Austria N=139 n (%)	Croatia N=134 n (%)	France N=115 n (%)	Germany N=263 n (%)	Greece N=217 n (%)	Italy N=589 n (%)	Norway N=137 n (%)	Poland N=157 n (%)	Portugal N=172 n (%)	Romania N=298 n (%)	Sweden N=219 n (%)	Switzerland N=417 n (%)
**Type of facility**												
Public	122 (87.8)	134 (100.0)	86 (74.8)	219 (83.3)	128 (59.0)	545 (92.5)	136 (99.3)	145 (92.4)	167 (97.1)	270 (90.6)	219 (100.0)	389 (93.3)
Private	17 (12.2)	0 (0.0)	29 (25.2)	44 (16.7)	89 (41.0)	44 (7.5)	1 (0.7)	12 (7.6)	5 (2.9)	28 (9.4)	0 (0.0)	28 (6.7)
**Professional qualification**												
General physician	0 (0.0)	0 (0.0)	1 (0.9)	0 (0.0)	12 (5.5)	6 (1.0)	0 (0.0)	2 (1.3)	2 (1.2)	8 (2.7)	0 (0.0)	0 (0.0)
Midwife	119 (85.6)	75 (56.0)	72 (62.6)	245 (93.2)	124 (57.1)	328 (55.7)	131 (95.6)	124 (79.0)	41 (23.8)	51 (17.1)	116 (53.0)	291 (69.8)
Nurse	7 (5.0)	22 (16.4)	21 (18.3)	8 (3.0)	19 (8.8)	102 (17.3)	5 (3.6)	11 (7.0)	42 (24.4)	148 (49.7)	4 (1.8)	93 (22.3)
Neonatologist	7 (5.0)	12 (9.0)	13 (11.3)	2 (0.8)	20 (9.2)	34 (5.8)	0 (0.0)	5 (3.2)	48 (27.9)	40 (13.4)	2 (0.9)	12 (2.9)
Obstetrician Gynaecologist	6 (4.3)	17 (12.7)	5 (4.3)	6 (2.3)	20 (9.2)	104 (17.7)	1 (0.7)	13 (8.3)	25 (14.5)	34 (11.4)	71 (32.4)	12 (2.9)
Registrar/medical resident [1]	0 (0.0)	8 (6.0)	3 (2.6)	2 (0.8)	22 (10.1)	15 (2.5)	0 (0.0)	2 (1.3)	14 (8.1)	17 (5.7)	26 (11.9)	9 (2.2)
**Gender (self-described)**												
Male	6 (4.3)	13 (9.7)	5 (4.3)	7 (2.7)	40 (18.4)	37 (6.3)	0 (0.0)	9 (5.7)	21 (12.2)	19 (6.4)	14 (6.4)	20 (4.8)
Female	129 (92.8)	114 (85.1)	110 (95.7)	249 (94.7)	166 (76.5)	531 (90.2)	135 (98.5)	144 (91.7)	131 (76.2)	273 (91.6)	204 (93.2)	391 (93.8)
Non-binary/gender fluid /agender/other	0 (0.0)	0 (0.0)	0 (0.0)	2 (0.8)	1 (0.5)	3 (0.5)	0 (0.0)	2 (1.3)	0 (0.0)	1 (0.3)	0 (0.0)	1 (0.2)
I prefer not to answer	4 (2.9)	0 (0.0)	0 (0.0)	5 (1.9)	10 (4.6)	14 (2.4)	0 (0.0)	2 (1.3)	0 (0.0)	4 (1.3)	0 (0.0)	4 (1.0)
Missing [2]	0 (0.0)	7 (5.2)	0 (0.0)	0 (0.0)	0 (0.0)	4 (0.7)	2 (1.5)	0 (0.0)	20 (11.6)	1 (0.3)	1 (0.5)	1 (0.2)
**Working experience (years)**												

<5	39 (28.1)	35 (26.1)	12 (10.4)	57 (21.7)	47 (21.7)	117 (19.9)	34 (24.8)	43 (27.4)	31 (18.0)	43 (14.4)	57 (26.0)	92 (22.1)
5-10	38 (27.3)	20 (14.9)	21 (18.3)	49 (18.6)	54 (24.9)	111 (18.8)	26 (19.0)	29 (18.5)	21 (12.2)	60 (20.1)	43 (19.6)	95 (22.8)
>10	62 (44.6)	72 (53.7)	82 (71.3)	157 (59.7)	116 (53.5)	358 (60.8)	75 (54.7)	85 (54.1)	100 (58.1)	194 (65.1)	118 (53.9)	230 (55.2)
Missing [2]	0 (0.0)	7 (5.2)	0 (0.0)	0 (0.0)	0 (0.0)	3 (0.5)	2 (1.5)	0 (0.0)	20 (11.6)	1 (0.3)	1 (0.5)	0 (0.0)
**Age (years)**												
20-29	35 (25.2)	36 (26.9)	15 (13.0)	51 (19.4)	36 (16.6)	84 (14.3)	13 (9.5)	51 (32.5)	17 (9.9)	26 (8.7)	13 (5.9)	75 (18.0)
30-39	48 (34.5)	28 (20.9)	37 (32.2)	67 (25.5)	69 (31.8)	184 (31.2)	50 (36.5)	34 (21.7)	46 (26.7)	71 (23.8)	66 (30.1)	152 (36.5)
40-49	34 (24.5)	31 (23.1)	34 (29.6)	68 (25.9)	70 (32.3)	164 (27.8)	35 (25.5)	27 (17.2)	35 (20.3)	106 (35.6)	56 (25.6)	100 (24.0)
50-59	16 (11.5)	25 (18.7)	26 (22.6)	65 (24.7)	32 (14.7)	122 (20.7)	24 (17.5)	35 (22.3)	36 (20.9)	83 (27.9)	56 (25.6)	71 (17.0)
60-69	6 (4.3)	7 (5.2)	3 (2.6)	12 (4.6)	10 (4.6)	29 (4.9)	11 (8.0)	9 (5.7)	18 (10.5)	11 (3.7)	27 (12.3)	18 (4.3)
≥70	0 (0.0)	0 (0.0)	0 (0.0)	0 (0.0)	0 (0.0)	2 (0.3)	2 (1.5)	1 (0.6)	0 (0.0)	0 (0.0)	0 (0.0)	1 (0.2)
Missing [2]	0 (0.0)	7 (5.2)	0 (0.0)	0 (0.0)	0 (0.0)	4 (0.7)	2 (1.5)	0 (0.0)	20 (11.6)	1 (0.3)	1 (0.5)	0 (0.0)
**Year of questionnaire completion**												
2021	2 (1.4)	107 (79.9)	115 (100.0)	254 (96.6)	0 (0.0)	285 (48.4)	132 (96.4)	144 (91.7)	140 (81.4)	290 (97.3)	210 (95.9)	326 (78.2)
2022	136 (97.8)	17 (12.7)	0 (0.0)	9 (3.4)	217 (100.0)	295 (50.1)	3 (2.2)	13 (8.3)	12 (7.0)	5 (1.7)	8 (3.7)	89 (21.3)
2023	0 (0.0)	0 (0.0)	0 (0.0)	0 (0.0)	0 (0.0)	2 (0.3)	0 (0.0)	0 (0.0)	0 (0.0)	0 (0.0)	0 (0.0)	0 (0.0)
Missing [2]	1 (0.7)	10 (7.5)	0 (0.0)	0 (0.0)	0 (0.0)	7 (1.2)	2 (1.5)	0 (0.0)	20 (11.6)	3 (1.0)	1 (0.5)	2 (0.5)

Notes: [1] A doctor in specialist training for Obstetrics and Gynecology or Neonatology; [2] Sociodemographic questions were placed at the end of the questionnaire.

Supplementary Table 5. Need for improvement in provision of care domain

	Overall	Austria	Croatia	France	Germany	Greece	Italy	Norway	Poland	Portugal	Romania	Sweden	Switzerland
**Quality Measures**	**N=4143**	**N=139**	**N=134**	**N=115**	**N=263**	**N=217**	**N=589**	**N=137**	**N=157**	**N=172**	**N=298**	**N=219**	**N=417**
	**n (%)**	**n (%)**	**n (%)**	**n (%)**	**n (%)**	**n (%)**	**n (%)**	**n (%)**	**n (%)**	**n (%)**	**n (%)**	**n (%)**	**n (%)**
Equipment and supplies for healthy women/newborns care[1]	1210 (29.2)	32 (23.0)	46 (34.3)	15 (13.0)	70 (26.6)	82 (37.8)	203 (34.5)	33 (24.1)	55 (35.0)	51 (29.7)	114 (38.3)	34 (15.5)	65 (15.6)
Guidelines and protocols for healthy women/newborns [1]	1664 (40.2)	58 (41.7)	71 (53.0)	47 (40.9)	99 (37.6)	114 (52.5)	279 (47.4)	20 (14.6)	68 (43.3)	71 (41.3)	131 (44.0)	40 (18.3)	130 (31.2)
Supportive supervision [1]	2540 (61.3)	104 (74.8)	84 (62.7)	70 (60.9)	215 (81.7)	135 (62.2)	396 (67.2)	66 (48.2)	111 (70.7)	109 (63.4)	160 (53.7)	95 (43.4)	230 (55.2)
Effective training on management of healthy women/newborns [1,2,3]	2748 (66.3)	113 (81.3)	84 (62.7)	95 (82.6)	200 (76.0)	135 (62.2)	410 (69.6)	94 (68.6)	113 (72.0)	99 (57.6)	198 (66.4)	133 (60.7)	306 (73.4)
Guidelines and protocols for emergencies	1403 (33.9)	43 (30.9)	77 (57.5)	34 (29.6)	100 (38.0)	117 (53.9)	237 (40.2)	17 (12.4)	71 (45.2)	69 (40.1)	135 (45.3)	33 (15.1)	111 (26.6)
Referral system for emergencies	1213 (29.3)	30 (21.6)	43 (32.1)	40 (34.8)	78 (29.7)	96 (44.2)	161 (27.3)	18 (13.1)	78 (49.7)	54 (31.4)	153 (51.3)	48 (21.9)	87 (20.9)
Effective training on management of emergencies [2,4]	2349 (56.7)	100 (71.9)	86 (64.2)	85 (73.9)	184 (70.0)	151 (69.6)	411 (69.8)	59 (43.1)	114 (72.6)	79 (45.9)	153 (51.3)	158 (72.1)	246 (59.0)
Systems to routinely monitor quality of care	2345 (56.6)	106 (76.3)	97 (72.4)	73 (63.5)	202 (76.8)	163 (75.1)	394 (66.9)	71 (51.8)	116 (73.9)	108 (62.8)	194 (65.1)	94 (42.9)	285 (68.3)
Weekly clinical meetings	2643 (63.8)	114 (82.0)	114 (85.1)	79 (68.7)	226 (85.9)	164 (75.6)	501 (85.1)	114 (83.2)	138 (87.9)	103 (59.9)	228 (76.5)	162 (74.0)	329 (78.9)
Maternal/neonatal deaths audits	2165 (52.3)	93 (66.9)	111 (82.8)	54 (47.0)	185 (70.3)	164 (75.6)	359 (61.0)	55 (40.1)	136 (86.6)	118 (68.6)	218 (73.2)	141 (64.4)	219 (52.5)

Notes: All Quality Measures in the domain of provision of care are directly based on WHO standards. [1] For case management of healthy women/newborns; [2] At least one training event in the last 3 years; [3] Only for maternal area: Partogram, fetal well-being, unnecessary caesarean section—only for neonatal area: breastfeeding promotion, skin-to-skin, standards precautions; [4] Only for maternal area: postpartum haemorrhage, eclampsia, shoulder dystocia, pregnant woman cardiovascular arrest—only neonatal area: newborn resuscitation.

Supplementary Table 6. Need for improvement in experience of care domain

	Overall	Austria	Croatia	France	Germany	Greece	Italy	Norway	Poland	Portugal	Romania	Sweden	Switzerland
**Quality Measures**	**N=4143**	**N=139**	**N=134**	**N=115**	**N=263**	**N=217**	**N=589**	**N=137**	**N=157**	**N=172**	**N=298**	**N=219**	**N=417**
	**n (%)**	**n (%)**	**n (%)**	**n (%)**	**n (%)**	**n (%)**	**n (%)**	**n (%)**	**n (%)**	**n (%)**	**n (%)**	**n (%)**	**n (%)**
Handover	1523 (36.8)	42 (30.2)	65 (48.5)	50 (43.5)	98 (37.3)	103 (47.5)	294 (49.9)	59 (43.1)	73 (46.5)	57 (33.1)	96 (32.2)	122 (55.7)	159 (38.1)
Communication with users	1566 (37.8)	51 (36.7)	78 (58.2)	24 (20.9)	110 (41.8)	131 (60.4)	348 (59.1)	35 (25.5)	98 (62.4)	70 (40.7)	146 (49.0)	99 (45.2)	113 (27.1)
Educational health materials for users	2192 (52.9)	66 (47.5)	95 (70.9)	73 (63.5)	155 (58.9)	163 (75.1)	421 (71.5)	83 (60.6)	104 (66.2)	104 (60.5)	205 (68.8)	119 (54.3)	213 (51.1)
Effective training on counselling/ communication [1]	2258 (54.5)	83 (59.7)	71 (53.0)	91 (79.1)	152 (57.8)	134 (61.8)	399 (67.7)	99 (72.3)	107 (68.2)	114 (66.3)	184 (61.7)	172 (78.5)	249 (59.7)
Labour companionship guaranteed	1651 (39.9)	26 (18.7)	81 (60.4)	39 (33.9)	72 (27.4)	124 (57.1)	228 (38.7)	83 (60.6)	108 (68.8)	94 (54.7)	260 (87.2)	117 (53.4)	138 (33.1)
Effective training on emotional support [1]	2399 (57.9)	80 (57.6)	82 (61.2)	87 (75.7)	141 (53.6)	139 (64.1)	424 (72.0)	108 (78.8)	121 (77.1)	124 (72.1)	229 (76.8)	179 (81.7)	288 (69.1)
Users' privacy guaranteed	1797 (43.4)	65 (46.8)	88 (65.7)	42 (36.5)	116 (44.1)	109 (50.2)	335 (56.9)	82 (59.9)	104 (66.2)	107 (62.2)	166 (55.7)	111 (50.7)	196 (47.0)
Consent request material aids [2]	2770 (66.9)	129 (92.8)	108 (80.6)	99 (86.1)	211 (80.2)	184 (84.8)	480 (81.5)	128 (93.4)	145 (92.4)	127 (73.8)	238 (79.9)	175 (79.9)	348 (83.5)
Effective training on informed consent [1]	2555 (61.7)	95 (68.3)	91 (67.9)	99 (86.1)	156 (59.3)	149 (68.7)	492 (83.5)	115 (83.9)	137 (87.3)	125 (72.7)	222 (74.5)	190 (86.8)	317 (76.0)
Effective training on pain relief practices [1,3]	2353 (56.8)	93 (66.9)	91 (67.9)	88 (76.5)	165 (62.7)	155 (71.4)	433 (73.5)	107 (78.1)	120 (76.4)	104 (60.5)	222 (74.5)	181 (82.6)	279 (66.9)

Notes: All Quality Measures in the domain of experience of care are directly based on WHO standards. [1] At least one training event in the last 3 years; [2] For maternal area: regular orientation sections for women during pregnancy, written/digital material for consent before ceasarean section, induction of labour - For neonatal area: regular orientation sections for women during pregnancy, written/digital material for consent before newborn Vitamin K administration, Newborn eye drops/ointment application; [3] For maternal area: pharmacological and non-pharmacological pain relief on labour - For neonatal area: prevention/ management of newborn's pain.

Supplementary Table 7. Need for improvement in availability of motivated and competent human and physical resources domain

	Overall	Austria	Croatia	France	Germany	Greece	Italy	Norway	Poland	Portugal	Romania	Sweden	Switzerland
**Quality Measures**	**N=4143**	**N=139**	**N=134**	**N=115**	**N=263**	**N=217**	**N=589**	**N=137**	**N=157**	**N=172**	**N=298**	**N=219**	**N=417**
	**n (%)**	**n (%)**	**n (%)**	**n (%)**	**n (%)**	**n (%)**	**n (%)**	**n (%)**	**n (%)**	**n (%)**	**n (%)**	**n (%)**	**n (%)**
Infrastructure for continuity of care [1]	1225 (29.6)	30 (21.6)	49 (36.6)	3 (2.6)	70 (26.6)	70 (32.3)	235 (39.9)	25 (18.2)	52 (33.1)	60 (34.9)	123 (41.3)	39 (17.8)	48 (11.5)
Infrastructure for emergencies	933 (22.5)	27 (19.4)	35 (26.1)	3 (2.6)	60 (22.8)	70 (32.3)	184 (31.2)	17 (12.4)	52 (33.1)	43 (25.0)	121 (40.6)	19 (8.7)	49 (11.8)
Equipment and supplies for emergencies	980 (23.7)	24 (17.3)	43 (32.1)	9 (7.8)	48 (18.3)	87 (40.1)	174 (29.5)	23 (16.8)	48 (30.6)	50 (29.1)	132 (44.3)	18 (8.2)	62 (14.9)
Tutoring organised during emergencies	2284 (55.1)	104 (74.8)	87 (64.9)	65 (56.5)	218 (82.9)	135 (62.2)	387 (65.7)	44 (32.1)	110 (70.1)	104 (60.5)	169 (56.7)	100 (45.7)	217 (52.0)
Staff number to ensure adequate care	2684 (64.8)	107 (77.0)	113 (84.3)	79 (68.7)	192 (73.0)	162 (74.7)	452 (76.7)	101 (73.7)	136 (86.6)	114 (66.3)	222 (74.5)	168 (76.7)	301 (72.2)
Clear definition of roles and responsibilities	1985 (47.9)	60 (43.2)	97 (72.4)	48 (41.7)	146 (55.5)	139 (64.1)	391 (66.4)	61 (44.5)	113 (72.0)	99 (57.6)	163 (54.7)	100 (45.7)	168 (40.3)
Existence of clinical data collection system	1759 (42.5)	52 (37.4)	74 (55.2)	51 (44.3)	136 (51.7)	127 (58.5)	362 (61.5)	55 (40.1)	96 (61.1)	59 (34.3)	98 (32.9)	84 (38.4)	217 (52.0)
Infrastructure to ensure privacy	1796 (43.4)	62 (44.6)	95 (70.9)	33 (28.7)	113 (43.0)	123 (56.7)	348 (59.1)	75 (54.7)	107 (68.2)	108 (62.8)	206 (69.1)	95 (43.4)	152 (36.5)
Existence of a quality of care improvement dedicated team	2274 (54.9)	101 (72.7)	112 (83.6)	85 (73.9)	190 (72.2)	156 (71.9)	489 (83.0)	63 (46.0)	121 (77.1)	111 (64.5)	198 (66.4)	85 (38.8)	245 (58.8)
Effective training covering rights of women/newborns [2]	2714 (65.5)	115 (82.7)	103 (76.9)	108 (93.9)	201 (76.4)	160 (73.7)	500 (84.9)	123 (89.8)	128 (81.5)	127 (73.8)	235 (78.9)	181 (82.6)	358 (85.9)

Notes: All Quality Measures in the domain of availability of motivated and competent human and physical resources are directly based on WHO standards. [1] For healthy women/newborns care; [2] At least one training event in the last 3 years.

Supplementary Table 8. Frequency of not adequate or happened during the COVID-19 pandemic in the organizational changes due to COVID-19 pandemic domain

	Overall	Austria	Croatia	France	Germany	Greece	Italy	Norway	Poland	Portugal	Romania	Sweden	Switzerland
**Quality Measures**	**N=4143**	**N=139**	**N=134**	**N=115**	**N=263**	**N=217**	**N=589**	**N=137**	**N=157**	**N=172**	**N=298**	**N=219**	**N=417**
	**n (%)**	**n (%)**	**n (%)**	**n (%)**	**n (%)**	**n (%)**	**n (%)**	**n (%)**	**n (%)**	**n (%)**	**n (%)**	**n (%)**	**n (%)**
Paths COVID-19 suspected/confirmed cases	1532 (37.0)	62 (44.6)	47 (35.1)	54 (47.0)	133 (50.6)	97 (44.7)	371 (63.0)	51 (37.2)	86 (54.8)	66 (38.4)	74 (24.8)	102 (46.6)	203 (48.7)
Availability of sufficient PPE	1326 (32.0)	47 (33.8)	35 (26.1)	58 (50.4)	127 (48.3)	75 (34.6)	293 (49.7)	43 (31.4)	87 (55.4)	40 (23.3)	84 (28.2)	122 (55.7)	172 (41.2)
Functioning and accessible hand hygiene stations	652 (15.7)	15 (10.8)	24 (17.9)	24 (20.9)	24 (9.1)	51 (23.5)	166 (28.2)	24 (17.5)	58 (36.9)	21 (12.2)	66 (22.1)	45 (20.5)	50 (12.0)
Updated guidelines	1550 (37.4)	73 (52.5)	48 (35.8)	56 (48.7)	144 (54.8)	104 (47.9)	348 (59.1)	37 (27.0)	90 (57.3)	68 (39.5)	90 (30.2)	94 (42.9)	203 (48.7)
Availability of nasopharyngeal swabs	1022 (24.7)	23 (16.5)	19 (14.2)	49 (42.6)	92 (35.0)	74 (34.1)	254 (43.1)	42 (30.7)	71 (45.2)	28 (16.3)	73 (24.5)	94 (42.9)	84 (20.1)
Effective training on COVID-19	1564 (37.8)	65 (46.8)	41 (30.6)	68 (59.1)	164 (62.4)	96 (44.2)	310 (52.6)	69 (50.4)	97 (61.8)	59 (34.3)	61 (20.5)	105 (47.9)	247 (59.2)
Closure or reduction of services	1809 (43.7)	78 (56.1)	69 (51.5)	51 (44.3)	116 (44.1)	115 (53.0)	320 (54.3)	60 (43.8)	107 (68.2)	105 (61.0)	164 (55.0)	149 (68.0)	240 (57.6)
Insufficient HW during pandemic	1941 (46.9)	93 (66.9)	85 (63.4)	47 (40.9)	139 (52.9)	157 (72.4)	302 (51.3)	78 (56.9)	131 (83.4)	74 (43.0)	186 (62.4)	166 (75.8)	257 (61.6)
Silencing (censorship) of staff	929 (22.4)	43 (30.9)	44 (32.8)	28 (24.3)	43 (16.3)	101 (46.5)	134 (22.8)	23 (16.8)	89 (56.7)	39 (22.7)	70 (23.5)	101 (46.1)	91 (21.8)
Limitations in QMNC due to COVID-19 [1]	50 (33.0)	62 (36.3)	36 (46.6)	66 (31.4)	152 (24.9)	151 (69.8)	227 (38.5)	44 (32.3)	92 (58.9)	86 (49.8)	163 (54.7)	122 (55.7)	115 (27.5)

Notes: [1] Frequency is calculated on seven indicators contributing to the same Quality Measure: Increased medicalization and/or limitations on companionship, restrictions on movements during labour, limitations on pain relief procedures, limitations on rooming-in practices without clinical indications, limitations on breastfeeding without clinical indications, limitations on skin to skin in absence of clear medical indications.
Abbreviations: HW = health workers; PPE = personal protective equipment; QMNC = quality of maternal and newborn care

Supplementary Table 9. Provision of care domain secondary analysis: need for significant improvement

Quality Measures	Overall N=4115 n (%)	Austria N=139 n (%)	Croatia N=134 n (%)	France N=115 n (%)	Germany N=262 n (%)	Greece N=215 n (%)	Italy N=582 n (%)	Norway N=137 n (%)	Poland N=157 n (%)	Portugal N=172 n (%)	Romania N=298 n (%)	Sweden N=219 n (%)	Switzerland N=417 n (%)
Equipment and supplies for healthy women/newborns care [1]	175 (4.3)	6 (4.3)	8 (6.0)	1 (0.9)	10 (3.8)	16 (7.4)	18 (3.1)	0 (0.0)	3 (1.9)	7 (4.1)	18 (6.0)	3 (1.4)	6 (1.4)
Guidelines and protocols for healthy women/newborns [1]	372 (9.0)	13 (9.4)	25 (18.7)	6 (5.2)	33 (12.6)	45 (20.9)	55 (9.5)	0 (0.0)	17 (10.8)	7 (4.1)	31 (10.4)	6 (2.7)	18 (4.3)
Supportive supervision [1]	1016 (24.7)	68 (48.9)	28 (20.9)	28 (24.3)	161 (61.5)	62 (28.8)	132 (22.7)	5 (3.6)	55 (35.0)	40 (23.3)	42 (14.1)	29 (13.2)	75 (18.0)
Effective training on management of healthy women/newborns[,2,3]	269 (6.5)	11 (7.9)	19 (14.2)	2 (1.7)	24 (9.2)	37 (17.2)	29 (5.0)	0 (0.0)	15 (9.6)	7 (4.1)	30 (10.1)	0 (0.0)	15 (3.6)
Guidelines and protocols for emergencies	214 (5.2)	10 (7.2)	7 (5.2)	4 (3.5)	9 (3.4)	21 (9.8)	25 (4.3)	1 (0.7)	10 (6.4)	11 (6.4)	46 (15.4)	8 (3.7)	10 (2.4)
Referral system for emergencies	981 (23.8)	56 (40.3)	42 (31.3)	31 (27.0)	118 (45.0)	108 (50.2)	133 (22.9)	22 (16.1)	60 (38.2)	33 (19.2)	84 (28.2)	23 (10.5)	110 (26.4)
Effective training on management of emergencies [2,4]	1816 (44.1)	96 (69.1)	78 (58.2)	44 (38.3)	188 (71.8)	115 (53.5)	344 (59.1)	75 (54.7)	126 (80.3)	70 (40.7)	129 (43.3)	110 (50.2)	213 (51.1)
Systems to routinely monitor quality of care	1093 (26.6)	47 (33.8)	68 (50.7)	18 (15.7)	84 (32.1)	122 (56.7)	148 (25.4)	17 (12.4)	108 (68.8)	66 (38.4)	122 (40.9)	67 (30.6)	59 (14.1)

Notes: All Quality Measures in the domain of provision of care are directly based on WHO standards. [1] For case management of healthy women/newborns; [2] At least one training event in the last 3 years; [3] Only for maternal area: Partogram, fetal well-being, unnecessary caesarean section—only for neonatal area: breastfeeding promotion, skin-to-skin, standards precautions; [4] Only for maternal area: postpartum haemorrhage, eclampsia, shoulder dystocia, pregnant woman cardiovascular arrest—only neonatal area: newborn resuscitation.

Supplementary Table 10. Experience of care domain secondary analysis: need for significant improvement

Quality Measures	Overall N=4115 n (%)	Austria N=139 n (%)	Croatia N=134 n (%)	France N=115 n (%)	Germany N=262 n (%)	Greece N=215 n (%)	Italy N=582 n (%)	Norway N=137 n (%)	Poland N=157 n (%)	Portugal N=172 n (%)	Romania N=298 n (%)	Sweden N=219 n (%)	Switzerland N=417 n (%)
Handover	280 (6.8)	6 (4.3)	13 (9.7)	8 (7.0)	23 (8.8)	16 (7.4)	55 (9.5)	9 (6.6)	12 (7.6)	10 (5.8)	10 (3.4)	26 (11.9)	21 (5.0)
Communication with users	210 (5.1)	9 (6.5)	13 (9.7)	2 (1.7)	22 (8.4)	32 (14.9)	33 (5.7)	1 (0.7)	15 (9.6)	4 (2.3)	17 (5.7)	15 (6.8)	5 (1.2)
Educational health materials for users	602 (14.6)	11 (7.9)	30 (22.4)	15 (13.0)	32 (12.2)	80 (37.2)	132 (22.7)	20 (14.6)	37 (23.6)	26 (15.1)	64 (21.5)	26 (11.9)	40 (9.6)
Effective training on counselling/ communication[1]	1406 (34.2)	53 (38.1)	27 (20.1)	74 (64.3)	95 (36.3)	77 (35.8)	249 (42.8)	67 (48.9)	74 (47.1)	68 (39.5)	94 (31.5)	123 (56.2)	164 (39.3)
Labour companionship guaranteed	735 (17.9)	5 (3.6)	37 (27.6)	10 (8.7)	13 (5.0)	72 (33.5)	77 (13.2)	46 (33.6)	43 (27.4)	27 (15.7)	186 (62.4)	62 (28.3)	40 (9.6)
Effective training on emotional support [1]	1595 (38.8)	50 (36.0)	39 (29.1)	66 (57.4)	87 (33.2)	86 (40.0)	309 (53.1)	70 (51.1)	86 (54.8)	90 (52.3)	141 (47.3)	133 (60.7)	193 (46.3)
Users' privacy guaranteed	445 (10.8)	13 (9.4)	26 (19.4)	8 (7.0)	22 (8.4)	28 (13.0)	63 (10.8)	22 (16.1)	38 (24.2)	25 (14.5)	60 (20.1)	24 (11.0)	40 (9.6)
Consent request material aids [2]	1322 (32.1)	88 (63.3)	43 (32.1)	47 (40.9)	142 (54.2)	76 (35.3)	187 (32.1)	73 (53.3)	99 (63.1)	45 (26.2)	124 (41.6)	81 (37.0)	175 (42.0)
Effective training on informed consent [1]	1757 (42.7)	59 (42.4)	50 (37.3)	80 (69.6)	87 (33.2)	86 (40.0)	355 (61.0)	87 (63.5)	112 (71.3)	87 (50.6)	125 (41.9)	142 (64.8)	244 (58.5)
Effective training on pain relief practices [1,3]	1496 (36.4)	55 (39.6)	52 (38.8)	62 (53.9)	95 (36.3)	93 (43.3)	275 (47.3)	73 (53.3)	80 (51.0)	69 (40.1)	146 (49.0)	127 (58.0)	172 (41.2)

Notes: All Quality Measures in the domain of experience of care are directly based on WHO standards. [1] At least one training event in the last 3 years; [2] For maternal area: regular orientation sections for women during pregnancy, written/digital material for consent before ceasarean section, induction of labour - For neonatal area: regular orientation sections for women during pregnancy, written/digital material for consent before newborn Vitamin K administration, Newborn eye drops/ointment application; [3] For maternal area: pharmacological and non-pharmacological pain relief on labour - For neonatal area: prevention/ management of newborn's pain.

Supplementary Table 11. Availability of motivated and competent human and physical resources domain secondary analysis: need for significant improvement

Quality Measures	Overall N=4115 n (%)	Austria N=139 n (%)	Croatia N=134 n (%)	France N=115 n (%)	Germany N=262 n (%)	Greece N=215 n (%)	Italy N=582 n (%)	Norway N=137 n (%)	Poland N=157 n (%)	Portugal N=172 n (%)	Romania N=298 n (%)	Sweden N=219 n (%)	Switzerland N=417 n (%)
Infrastructure for continuity of care [1]	164 (4.0)	8 (5.8)	6 (4.5)	0 (0.0)	13 (5.0)	11 (5.1)	27 (4.6)	3 (2.2)	6 (3.8)	3 (1.7)	15 (5.0)	7 (3.2)	2 (0.5)
Infrastructure for emergencies	95 (2.3)	2 (1.4)	2 (1.5)	0 (0.0)	12 (4.6)	11 (5.1)	15 (2.6)	2 (1.5)	3 (1.9)	2 (1.2)	14 (4.7)	1 (0.5)	1 (0.2)
Equipment and supplies for emergencies	119 (2.9)	2 (1.4)	6 (4.5)	0 (0.0)	6 (2.3)	18 (8.4)	13 (2.2)	0 (0.0)	5 (3.2)	4 (2.3)	18 (6.0)	2 (0.9)	4 (1.0)
Tutoring organised during emergencies	853 (20.7)	64 (46.0)	29 (21.6)	15 (13.0)	149 (56.9)	55 (25.6)	112 (19.2)	6 (4.4)	58 (36.9)	38 (22.1)	46 (15.4)	17 (7.8)	63 (15.1)
Staff number to ensure adequate care	1276 (31.0)	56 (40.3)	72 (53.7)	44 (38.3)	99 (37.8)	86 (40.0)	204 (35.1)	41 (29.9)	90 (57.3)	50 (29.1)	84 (28.2)	81 (37.0)	131 (31.4)
Clear definition of roles and responsibilities	617 (15.0)	19 (13.7)	50 (37.3)	8 (7.0)	34 (13.0)	68 (31.6)	156 (26.8)	11 (8.0)	43 (27.4)	30 (17.4)	34 (11.4)	25 (11.4)	30 (7.2)
Existence of clinical data collection system	356 (8.7)	17 (12.2)	12 (9.0)	6 (5.2)	30 (11.5)	40 (18.6)	92 (15.8)	4 (2.9)	15 (9.6)	10 (5.8)	13 (4.4)	10 (4.6)	38 (9.1)
Infrastructure to ensure privacy	641 (15.6)	18 (12.9)	48 (35.8)	9 (7.8)	33 (12.6)	45 (20.9)	120 (20.6)	29 (21.2)	52 (33.1)	32 (18.6)	102 (34.2)	33 (15.1)	26 (6.2)
Existence of a quality of care improvement dedicated team	1063 (25.8)	53 (38.1)	75 (56.0)	29 (25.2)	113 (43.1)	96 (44.7)	266 (45.7)	18 (13.1)	72 (45.9)	30 (17.4)	79 (26.5)	21 (9.6)	78 (18.7)
Effective training covering rights of women/newborns[2]	2050 (49.8)	91 (65.5)	67 (50.0)	95 (82.6)	148 (56.5)	104 (48.4)	391 (67.2)	93 (67.9)	111 (70.7)	88 (51.2)	148 (49.7)	140 (63.9)	299 (71.7)

Notes: All Quality Measures in the domain of availability of motivated and competent human and physical resources are directly based on WHO standards. [1] for healthy women/newborns care; 2 At least one training event in the last 3 years.

Supplementary Table 12. Organizational changes due to COVID-19 pandemic domain secondary analysis: never adequate independently from pandemic

Quality Measures	Overall N=4115 n (%)	Austria N=139 n (%)	Croatia N=134 n (%)	France N=115 n (%)	Germany N=262 n (%)	Greece N=215 n (%)	Italy N=582 n (%)	Norway N=137 n (%)	Poland N=157 n (%)	Portugal N=172 n (%)	Romania N=298 n (%)	Sweden N=219 n (%)	Switzerland N=417 n (%)
Paths COVID-19 suspected/confirmed cases	102 (2.5)	1 (0.7)	8 (6.0)	4 (3.5)	9 (3.4)	11 (5.1)	13 (2.2)	0 (0.0)	16 (10.2)	2 (1.2)	5 (1.7)	4 (1.8)	13 (3.1)
Availability of sufficient PPE	80 (1.9)	1 (0.7)	4 (3.0)	0 (0.0)	9 (3.4)	8 (3.7)	11 (1.9)	0 (0.0)	11 (7.0)	1 (0.6)	9 (3.0)	3 (1.4)	6 (1.4)
Functioning and accessible hand hygiene stations	67 (1.6)	2 (1.4)	3 (2.2)	2 (1.7)	1 (0.4)	7 (3.3)	14 (2.4)	1 (0.7)	11 (7.0)	0 (0.0)	9 (3.0)	6 (2.7)	0 (0.0)
Updated guidelines	282 (6.9)	19 (13.7)	9 (6.7)	7 (6.1)	50 (19.1)	30 (14.0)	38 (6.5)	3 (2.2)	21 (13.4)	2 (1.2)	15 (5.0)	12 (5.5)	39 (9.4)
Availability of nasopharyngeal swabs	107 (2.6)	1 (0.7)	5 (3.7)	2 (1.7)	8 (3.1)	11 (5.1)	13 (2.2)	5 (3.6)	15 (9.6)	0 (0.0)	16 (5.4)	6 (2.7)	9 (2.2)
Effective training on COVID-19	381 (9.3)	16 (11.5)	12 (9.0)	21 (18.3)	53 (20.2)	17 (7.9)	45 (7.7)	13 (9.5)	36 (22.9)	12 (7.0)	12 (4.0)	18 (8.2)	83 (19.9)
Closure or reduction of services	189 (4.6)	14 (10.1)	5 (3.7)	5 (4.3)	21 (8.0)	5 (2.3)	24 (4.1)	4 (2.9)	5 (3.2)	3 (1.7)	8 (2.7)	34 (15.5)	48 (11.5)
Insufficient HW during pandemic	771 (18.7)	38 (27.3)	33 (24.6)	28 (24.3)	67 (25.6)	61 (28.4)	99 (17.0)	37 (27.0)	63 (40.1)	23 (13.4)	54 (18.1)	67 (30.6)	121 (29.0)
Silencing (censorship) of staff	435 (10.6)	19 (13.7)	25 (18.7)	14 (12.2)	27 (10.3)	46 (21.4)	46 (7.9)	8 (5.8)	43 (27.4)	21 (12.2)	23 (7.7)	76 (34.7)	34 (8.2)
Limitations in QMNC due to COVID-19 [1]	8.3%	5.8%	14.5%	6.0%	5.7%	22.7%	6.6%	10.2%	20.8%	5.7%	18.0%	15.1%	6.7%

Notes: [1] Frequency is calculated on seven indicators contributing to the same Quality Measure: Increased medicalisation and/or limitations on companionship, restrictions on movements during labour, limitations on pain relief procedures, limitations on rooming-in practices without clinical indications, limitations on breastfeeding without clinical indications, limitations on skin to skin in absence of clear medical indications.
Abbreviations: HW = health workers; PPE = personal protective equipment; QMNC = quality of maternal and newborn care

Supplementary Table 13. Sensitivity analysis - characteristics of respondent

	Overall N=3104<br>n (%)
**Country**	
Italy	585 (18.8)
Switzerland	416 (13.4)
Romania	273 (8.8)
Germany	262 (8.4)
Sweden	218 (7.0)
Greece	217 (7.0)
Poland	157 (5.1)
Portugal	152 (4.9)
Austria	139 (4.5)
Norway	135 (4.3)
Croatia	127 (4.1)
France	115 (3.7)
Other [1]	308 (9.9)
**Type of facility**	
Public	2794 (90.0)
Private	310 (10.0)
**Professional qualification**	
Midwife	1873 (60.3)
Nurse	478 (15.4)
Obstetrician Gynaecologist	383 (12.3)
Neonatologist	202 (6.5)
Registrar/medical resident [2]	136 (4.4)
General physician	32 (1.0)
**Gender (self-described)**	
Female	2836 (91.4)
Male	203 (6.5)
I prefer not to answer	52 (1.7)
Non-binary/gender fluid /agender/other	13 (0.4)
**Working experience (years)**	
>10	1829 (58.9)
10 - 5	604 (19.5)
<5	671 (21.6)
**Age (years)**	
20-29	501 (16.1)
30-39	932 (30.0)
40-49	831 (26.8)
50-59	649 (20.9)
60-69	185 (6.0)
≥70	6 (0.2)
**Year of questionnaire completion**	
2021	2160 (69.6)
2022	929 (29.9)
2023	2 (0.1)
Missing	13 (0.4)

Notes: [1] Other countries: Lithuania 75 (2.4), Slovenia 64 (2.1), Luxembourg 48 (1.5), Latvia 27 (0.9%), Spain 23 (0.7%), Bosnia-Herzegovina 12 (0.4%), Ireland 5 (0.2%), Montenegro 4 (0.1%), Serbia 4 (0.1%), UK 4 (0.1%), Cyprus 1 (0.0%), Denmark 1 (0.0%), Macedonia 1 (0.0%), Malta 1 (0.0%), Tajikistan 1 (0.0%), Ukraine 1 (0.0%), missing country 36 (1.2); [2] A doctor in specialist training for Obstetrics and Gynaecology or Neonatology.

Supplementary Table 14. Sensitivity analysis: Provision of care domain - frequency of need for improvement

	Overall	Austria	Croatia	France	Germany	Greece	Italy	Norway	Poland	Portugal	Romania	Sweden	Switzerland
**Quality Measures**	**N=3104**	**N=139**	**N=127**	**N=115**	**N=262**	**N=217**	**N=585**	**N=135**	**N=157**	**N=152**	**N=273**	**N=218**	**N=416**
	**n (%)**	**n (%)**	**n (%)**	**n (%)**	**n (%)**	**n (%)**	**N (%)**	**n (%)**	**n (%)**	**n (%)**	**n (%)**	**n (%)**	**n (%)**
Equipment and supplies for healthy women/newborns care[1]	860 (27.7)	32 (23.0)	45 (35.4)	15 (13.0)	70 (26.7)	82 (37.8)	202 (34.5)	33 (24.4)	55 (35.0)	44 (28.9)	108 (39.6)	34 (15.6)	65 (15.6)
Guidelines and protocols for healthy women/newborns [1]	1219 (39.3)	58 (41.7)	68 (53.5)	47 (40.9)	98 (37.4)	114 (52.5)	277 (47.4)	20 (14.8)	68 (43.3)	60 (39.5)	125 (45.8)	40 (18.3)	130 (31.2)
Supportive supervision [1]	1908 (61.5)	104 (74.8)	81 (63.8)	70 (60.9)	214 (81.7)	135 (62.2)	393 (67.2)	66 (48.9)	111 (70.7)	93 (61.2)	152 (55.7)	95 (43.6)	230 (55.3)
Effective training on management of healthy women/newborns [1,2,3]	2136 (68.8)	113 (81.3)	80 (63.0)	95 (82.6)	199 (76.0)	135 (62.2)	410 (70.1)	94 (69.6)	113 (72.0)	86 (56.6)	178 (65.2)	133 (61.0)	306 (73.6)
Guidelines and protocols for emergencies [4]	1138 (36.7)	43 (30.9)	74 (58.3)	34 (29.6)	100 (38.2)	117 (53.9)	236 (40.3)	17 (12.6)	71 (45.2)	61 (40.1)	125 (45.8)	33 (15.1)	111 (26.7)
Referral system for emergencies [4]	960 (30.9)	30 (21.6)	42 (33.1)	40 (34.8)	78 (29.8)	96 (44.2)	161 (27.5)	18 (13.3)	78 (49.7)	47 (30.9)	137 (50.2)	48 (22.0)	87 (20.9)
Effective training on management of emergencies [2,4,5]	1982 (63.9)	100 (71.9)	81 (63.8)	85 (73.9)	183 (69.8)	151 (69.6)	410 (70.1)	59 (43.7)	114 (72.6)	70 (46.1)	136 (49.8)	158 (72.5)	245 (58.9)
Systems to routinely monitor quality of care	2064 (66.5)	106 (76.3)	94 (74.0)	73 (63.5)	201 (76.7)	163 (75.1)	394 (67.4)	71 (52.6)	116 (73.9)	99 (65.1)	177 (64.8)	94 (43.1)	285 (68.5)
Weekly clinical meetings	2481 (79.9)	114 (82.0)	111 (87.4)	79 (68.7)	225 (85.9)	164 (75.6)	500 (85.5)	113 (83.7)	138 (87.9)	99 (65.1)	207 (75.8)	162 (74.3)	329 (79.1)
Maternal/neonatal deaths audits	2023 (65.2)	93 (66.9)	108 (85.0)	54 (47.0)	184 (70.2)	164 (75.6)	358 (61.2)	54 (40.0)	136 (86.6)	115 (75.7)	195 (71.4)	141 (64.7)	219 (52.6)

Notes: All Quality Measures in the domain of provision of care are directly based on WHO standards. 1 for case management of healthy women/newborns; 2 At least one training event in the last 3 years; 3 Only for maternal area: Partogram, fetal well-being, unnecessary caesarean section—only for neonatal area: breastfeeding promotion, skin-to-skin, standards precautions; 4 for case management of emergencies; 5 Only for maternal area: postpartum haemorrhage, eclampsia, shoulder dystocia, pregnant woman cardiovascular arrest—only neonatal area: newborn resuscitation.

Supplementary Table 15. Sensitivity analysis: Experience of care domain - frequency of need for improvement

	Overall	Austria	Croatia	France	Germany	Greece	Italy	Norway	Poland	Portugal	Romania	Sweden	Switzerland
**Quality Measures**	**N=3104**	**N=139**	**N=127**	**N=115**	**N=262**	**N=217**	**N=585**	**N=135**	**N=157**	**N=152**	**N=273**	**N=218**	**N=416**
	**n (%)**	**n (%)**	**n (%)**	**n (%)**	**n (%)**	**n (%)**	**n (%)**	**n (%)**	**n (%)**	**n (%)**	**n (%)**	**n (%)**	**n (%)**
Handover	1334 (43.0)	42 (30.2)	63 (49.6)	50 (43.5)	97 (37.0)	103 (47.5)	294 (50.3)	58 (43.0)	73 (46.5)	54 (35.5)	89 (32.6)	122 (56.0)	159 (38.2)
Communication with users	1409 (45.4)	51 (36.7)	76 (59.8)	24 (20.9)	110 (42.0)	131 (60.4)	348 (59.5)	35 (25.9)	98 (62.4)	64 (42.1)	133 (48.7)	99 (45.4)	113 (27.2)
Educational health materials for users	1960 (63.1)	66 (47.5)	93 (73.2)	73 (63.5)	155 (59.2)	163 (75.1)	421 (72.0)	82 (60.7)	104 (66.2)	97 (63.8)	188 (68.9)	119 (54.6)	213 (51.2)
Effective training on counseling/ communication [1]	2026 (65.3)	83 (59.7)	71 (55.9)	91 (79.1)	151 (57.6)	134 (61.8)	399 (68.2)	98 (72.6)	107 (68.2)	106 (69.7)	164 (60.1)	172 (78.9)	249 (59.9)
Labour companionship guaranteed	1490 (48.0)	26 (18.7)	80 (63.0)	39 (33.9)	72 (27.5)	124 (57.1)	228 (39.0)	82 (60.7)	108 (68.8)	88 (57.9)	236 (86.4)	117 (53.7)	138 (33.2)
Effective training on emotional support [1]	2193 (70.7)	80 (57.6)	81 (63.8)	87 (75.7)	140 (53.4)	139 (64.1)	424 (72.5)	107 (79.3)	121 (77.1)	118 (77.6)	206 (75.5)	179 (82.1)	288 (69.2)
Users' privacy guaranteed	1654 (53.3)	65 (46.8)	86 (67.7)	42 (36.5)	115 (43.9)	109 (50.2)	335 (57.3)	81 (60.0)	104 (66.2)	100 (65.8)	151 (55.3)	111 (50.9)	196 (47.1)
Consent request material aids [2]	2565 (82.6)	129 (92.8)	105 (82.7)	99 (86.1)	210 (80.2)	184 (84.8)	480 (82.1)	127 (94.1)	145 (92.4)	121 (79.6)	218 (79.9)	175 (80.3)	348 (83.7)
Effective training on informed consent [1]	2389 (77.0)	95 (68.3)	89 (70.1)	99 (86.1)	155 (59.2)	149 (68.7)	492 (84.1)	114 (84.4)	137 (87.3)	119 (78.3)	200 (73.3)	190 (87.2)	317 (76.2)
Effective training on pain relief practices [1,3]	2219 (71.5)	93 (66.9)	89 (70.1)	88 (76.5)	164 (62.6)	155 (71.4)	433 (74.0)	106 (78.5)	120 (76.4)	101 (66.4)	200 (73.3)	181 (83.0)	279 (67.1)

Notes: All Quality Measures in the domain of experience of care are directly based on WHO standards. 1 At least one training event in the last 3 years; 2 For maternal area: regular orientation sections for women during pregnancy, written/digital material for consent before ceasarean section, induction of labour - For neonatal area: regular orientation sections for women during pregnancy, written/digital material for consent before newborn Vitamin K administration, Newborn eye drops/ointment application; 3 For maternal area: pharmacological and non-pharmacological pain relief on labour - For neonatal area: prevention/ management of newborn's pain.

Supplementary Table 16. Sensitivity analysis: Availability of motivated and competent human and physical resources domain - frequency of need for improvement

	Overall	Austria	Croatia	France	Germany	Greece	Italy	Norway	Poland	Portugal	Romania	Sweden	Switzerland
**Quality Measures**	**N=3104**	**N=139**	**N=127**	**N=115**	**N=262**	**N=217**	**N=585**	**N=135**	**N=157**	**N=152**	**N=273**	**N=218**	**N=416**
	**n (%)**	**n (%)**	**n (%)**	**n (%)**	**n (%)**	**n (%)**	**n (%)**	**n (%)**	**n (%)**	**n (%)**	**n (%)**	**n (%)**	**n (%)**
Infrastructure for continuity of care [1]	858 (27.6)	30 (21.6)	48 (37.8)	3 (2.6)	69 (26.3)	70 (32.3)	235 (40.2)	25 (18.5)	52 (33.1)	51 (33.6)	115 (42.1)	39 (17.9)	48 (11.5)
Infrastructure for emergencies	727 (23.4)	27 (19.4)	34 (26.8)	3 (2.6)	59 (22.5)	70 (32.3)	184 (31.5)	17 (12.6)	52 (33.1)	37 (24.3)	112 (41.0)	19 (8.7)	49 (11.8)
Equipment and supplies for emergencies	774 (24.9)	24 (17.3)	43 (33.9)	9 (7.8)	47 (17.9)	87 (40.1)	174 (29.7)	23 (17.0)	48 (30.6)	46 (30.3)	123 (45.1)	18 (8.3)	62 (14.9)
Tutoring organised during emergencies	1882 (60.6)	104 (74.8)	85 (66.9)	65 (56.5)	217 (82.8)	135 (62.2)	386 (66.0)	44 (32.6)	110 (70.1)	93 (61.2)	157 (57.5)	100 (45.9)	217 (52.2)
Staff number to ensure adequate care	2320 (74.7)	107 (77.0)	109 (85.8)	79 (68.7)	191 (72.9)	162 (74.7)	452 (77.3)	101 (74.8)	136 (86.6)	106 (69.7)	202 (74.0)	168 (77.1)	301 (72.4)
Clear definition of roles and responsibilities	1730 (55.7)	60 (43.2)	93 (73.2)	48 (41.7)	146 (55.7)	139 (64.1)	391 (66.8)	61 (45.2)	113 (72.0)	91 (59.9)	150 (54.9)	100 (45.9)	168 (40.4)
Existence of clinical data collection system	1550 (49.9)	52 (37.4)	71 (55.9)	51 (44.3)	136 (51.9)	127 (58.5)	361 (61.7)	55 (40.7)	96 (61.1)	54 (35.5)	90 (33.0)	84 (38.5)	217 (52.2)
Infrastructure to ensure privacy	1646 (53.0)	62 (44.6)	93 (73.2)	33 (28.7)	112 (42.7)	123 (56.7)	348 (59.5)	74 (54.8)	107 (68.2)	101 (66.4)	188 (68.9)	95 (43.6)	152 (36.5)
Existence of a quality of care improvement dedicated team	2137 (68.8)	101 (72.7)	110 (86.6)	85 (73.9)	189 (72.1)	156 (71.9)	488 (83.4)	63 (46.7)	121 (77.1)	107 (70.4)	179 (65.6)	85 (39.0)	245 (58.9)
Effective training covering rights of women/newborns [2]	2557 (82.4)	115 (82.7)	100 (78.7)	108 (93.9)	200 (76.3)	160 (73.7)	499 (85.3)	122 (90.4)	128 (81.5)	123 (80.9)	211 (77.3)	181 (83.0)	358 (86.1)

Notes: All Quality Measures in the domain of Availability of motivated and competent human and physical resources are directly based on WHO standards. 1 for healthy women/newborns care; 2 At least one training event in the last 3 years.

Supplementary Table 17. Sensitivity analysis: Organizational changes due to COVID-19 pandemic domain - frequency of not adequate or happened during the COVID-19 pandemic

	Overall	Austria	Croatia	France	Germany	Greece	Italy	Norway	Poland	Portugal	Romania	Sweden	Switzerland
**Quality Measures**	**N=3104**	**N=139**	**N=127**	**N=115**	**N=262**	**N=217**	**N=585**	**N=135**	**N=157**	**N=152**	**N=273**	**N=218**	**N=416**
	**n (%)**	**n (%)**	**n (%)**	**n (%)**	**n (%)**	**n (%)**	**n (%)**	**n (%)**	**n (%)**	**n (%)**	**n (%)**	**n (%)**	**n (%)**
Paths COVID-19 suspected/confirmed cases	1454 (46.8)	62 (44.6)	47 (37.0)	54 (47.0)	132 (50.4)	97 (44.7)	370 (63.2)	50 (37.0)	86 (54.8)	64 (42.1)	66 (24.2)	102 (46.8)	203 (48.8)
Availability of sufficient PPE	1255 (40.4)	47 (33.8)	34 (26.8)	58 (50.4)	126 (48.1)	75 (34.6)	293 (50.1)	42 (31.1)	87 (55.4)	38 (25.0)	76 (27.8)	122 (56.0)	172 (41.3)
Functioning and accessible hand hygiene stations	603 (19.4)	15 (10.8)	22 (17.3)	24 (20.9)	24 (9.2)	51 (23.5)	166 (28.4)	24 (17.8)	58 (36.9)	20 (13.2)	59 (21.6)	45 (20.6)	50 (12.0)
Updated guidelines	1470 (47.4)	73 (52.5)	47 (37.0)	56 (48.7)	144 (55.0)	104 (47.9)	348 (59.5)	37 (27.4)	90 (57.3)	66 (43.4)	81 (29.7)	94 (43.1)	203 (48.8)
Availability of nasopharyngeal swabs	962 (31.0)	23 (16.5)	18 (14.2)	49 (42.6)	92 (35.1)	74 (34.1)	254 (43.4)	42 (31.1)	71 (45.2)	26 (17.1)	65 (23.8)	94 (43.1)	84 (20.2)
Effective training on COVID-19	1493 (48.1)	65 (46.8)	40 (31.5)	68 (59.1)	164 (62.6)	96 (44.2)	310 (53.0)	69 (51.1)	97 (61.8)	58 (38.2)	53 (19.4)	105 (48.2)	247 (59.4)
Closure or reduction of services	1730 (55.7)	78 (56.1)	67 (52.8)	51 (44.3)	116 44.3)	115 (53.0)	320 (54.7)	60 (44.4)	107 (68.2)	104 (68.4)	153 (56.0)	149 (68.3)	240 (57.7)
Insufficient HW during pandemic	1846 (59.5)	93 (66.9)	83 (65.4)	47 (40.9)	138 (52.7)	157 (72.4)	302 (51.6)	78 (57.8)	131 (83.4)	73 (48.0)	165 (60.4)	166 (76.1)	257 (61.8)
Silencing (censorship) of staff	877 (28.3)	43 (30.9)	43 (33.9)	28 (24.3)	43 (16.4)	101 (46.5)	134 (22.9)	23 (17.0)	89 (56.7)	38 (25.0)	58 (21.2)	101 (46.3)	91 (21.9)
Limitations in QMNC due to COVID-19 [1]	353 (36.3)	432 (48.6)	253 (31.4)	457 (24.9)	1061 (69.8)	1585 (38.7)	310 (32.8)	647 (58.9)	593 (55.7)	1078 (56.4)	854 (55.9)	804 (27.6)	353 (36.3)

Notes: 1 Frequency is calculated on seven indicators contributing to the same Quality Measure: Increased medicalization and/or limitations on companionship, restrictions on movements during labour, limitations on pain relief procedures, limitations on rooming-in practices without clinical indications, limitations on breastfeeding without clinical indications, limitations on skin to skin in absence of clear medical indications.

Abbreviations: HW = health workers; PPE = personal protective equipment; QMNC = quality of maternal and newborn care

Supplementary Table 18. QMNC Index by country and by domain

	N	QMNC Index	QMNC Index by domain			
		Median (IQR)	Provision of care Median (IQR)	Experience of care Median (IQR)	Availability of motivated and competent human and physical resources Median (IQR)	Organizational changes due to COVID-19 pandemic Median (IQR)
**TOTAL**	3195	260.24 (208.24; 308.93)	65.00 (48.33; 80.00)	55.00 (40.00; 72.50)	65.00 (50.00; 80.00)	77.86 (62.86; 89.29)
**Country**						
Austria	139	259.94 (219.52; 306.37)	60.00 (43.96; 75.83)	63.75 (49.38; 80.00)	65.00 (50.00; 80.00)	76.43 (66.79; 87.86)
Croatia	130	245.48 (190.57; 298.94)	62.08 (40.21; 73.33)	55.00 (38.12; 70.00)	55.00 (35.00; 70.00)	82.50 (67.86; 90.00)
France	115	266.37 (225.83; 307.08)	67.08 (51.67; 80.00)	55.00 (42.50; 65.00)	70.00 (60.00; 80.00)	78.57 (63.57; 88.57)
Germany	262	259.78 (210.76; 303.41)	56.67 (43.33; 72.50)	65.00 (47.50; 80.00)	63.75 (50.00; 75.00)	77.14 (64.29; 88.57)
Greece	217	222.50 (162.56; 314.82)	51.67 (31.67; 79.17)	50.00 (30.00; 77.50)	55.00 (35.00; 80.00)	73.57 (55.00; 90.00)
Italy	586	252.68 (203.41; 292.59)	63.96 (47.19; 78.33)	52.50 (37.50; 70.00)	60.00 (45.00; 75.00)	75.00 (62.32; 87.14)
Norway	135	277.86 (244.32; 308.30)	76.25 (65.42; 85.42)	50.00 (35.00; 63.75)	75.00 (60.00; 80.00)	84.29 (68.93; 90.00)
Poland	157	210.60 (155.71; 273.57)	53.75 (33.75; 70.42)	45.00 (30.00; 61.25)	55.00 (40.00; 70.00)	62.86 (48.57; 82.14)
Portugal	153	271.43 (213.81; 308.33)	67.50 (47.50; 85.00)	55.00 (40.00; 67.50)	65.00 (50.00; 75.00)	81.43 (70.00; 90.00)
Romania	273	256.49 (207.80; 314.94)	65.00 (45.00; 82.50)	50.00 (35.00; 70.00)	65.00 (50.00; 80.00)	84.29 (72.86; 92.14)
Sweden	218	261.34 (216.32; 315.13)	75.00 (58.44; 87.81)	47.50 (32.50; 66.88)	75.00 (60.00; 85.00)	70.00 (56.43; 82.86)
Switzerland	417	277.44 (233.57; 320.36)	70.00 (56.67; 83.75)	60.00 (45.00; 80.00)	70.00 (60.00; 80.00)	77.14 (65.00; 88.57)

Notes: Overall QMNC Index pairwise comparisons between domains were significant (adj p<0.001) except for the comparison provision vs availability of human and physical resources (adj p=0.061).
Abbreviations: IQR = interquartile range; QMNC = quality of maternal and newborn care.

Supplementary Table 19. Pairwise comparisons across four domains, overall and by country

		Pairwise comparisons					
		Experience vs Provision	Experience vs Resources	Experience vs COVID-19	Provision vs Resources	Provision vs COVID-19	Resources vs COVID-19
	n	Adj p-value	Adj p-value	Adj p-value	Adj p-value	Adj p-value	Adj p-value
Overall	3195	<0.001	<0.001	<0.001	0.061	<0.001	<0.001
**Country**							
Austria	139	0.403	>0.99	<0.001	0.080	<0.001	<0.001
Croatia	130	0.570	>0.99	<0.001	0.068	<0.001	<0.001
France	115	<0.001	<0.001	<0.001	0.099	<0.001	<0.001
Germany	262	<0.001	0.151	<0.001	0.001	<0.001	<0.001
Greece	217	0.750	<0.001	<0.001	<0.001	<0.001	<0.001
Italy	586	<0.001	<0.001	<0.001	<0.001	<0.001	<0.001
Norway	135	<0.001	<0.001	<0.001	<0.001	0.044	<0.001
Poland	157	<0.001	<0.001	<0.001	>0.99	<0.001	<0.001
Portugal	153	<0.001	<0.001	<0.001	0.296	<0.001	<0.001
Romania	273	<0.001	<0.001	<0.001	>0.99	<0.001	<0.001
Sweden	218	<0.001	<0.001	<0.001	>0.99	0.048	0.112
Switzerland	417	<0.001	<0.001	<0.001	>0.99	<0.001	<0.001

Supplementary Table 20. Multivariate linear regression model with QMNC Index as outcome

	β estimates (95% CI)	p-value
**Country**		
Austria	34.23 (17.86; 50.60)	<0.001
Croatia	0.49 (-13.14; 14.12)	0.944
France	11.83 (0.25; 23.41)	0.045
Germany	17.56 (7.96; 27.16)	<0.001
Greece	-12.62 (-30.48; 5.23)	0.166
Italy	Reference	
Norway	41.81 (31.32; 52.31)	<0.001
Poland	-23.68 (-36.46; -10.90)	<0.001
Portugal	6.96 (-4.92; 18.83)	0.251
Romania	-0.67 (-11.52; 10.19)	0.904
Sweden	17.73 (7.75; 27.71)	0.001
Switzerland	33.23 (25.41; 41.06)	<0.001
Other [1]	23.20 (13.76; 32.65)	<0.001
**Gender**		
Male	12.61 (1.86; 23.36)	0.021
Female	Reference	
Non-binary/gender fluid /agender/other	-32.72 (-75.53; 10.09)	0.134
I prefer not to answer	-1.17 (-25.66; 23.33)	0.926
**Professional qualification**		
General physician	66.84 (39.23; 94.45)	<0.001
Midwife	Reference	
Nurse	16.84 (9.47; 24.22)	<0.001
Neonatologist physician	27.94 (18.12; 37.76)	<0.001
Obstetrics and gynaecology physician	23.17 (14.86; 31.49)	<0.001
Registrar/resident	23.96 (10.46; 37.46)	0.001
**Type of facility**		

Public	Reference	
Private	30.07 (21.24; 38.91)	<0.001
**Working experience (years)**		
<5	-30.29 (-36.42; -24.17)	<0.001
5-10	-25.60 (-31.8; -19.39)	<0.001
>10	Reference	
**Emergency phase of the COVID-19 pandemic [2]**		
During emergency phase (before 27 April 2022)	Reference	
After emergency phase (after 27 April 2022)	-9.86 (-23.19; 3.48)	0.147
**Intercept**	246.32 (239.93; 252.70)	<0.001

Note: n=3104; robust estimates of standard errors have been calculated. [1] Countries with <100 respondents (Bosnia-Herzegovina, Cyprus, Denmark, Ireland, Macedonia, Malta, Montenegro, Latvia, Lithuania, Luxembourg, Serbia, Slovenia, Spain, Tajikistan, UK, and Ukraine) were considered for this analysis as a single category; [2] on 27 April 2022 the European Union (EU) declared that the EU was moving out of the emergency phase of the COVID-19 pandemic (Communication from the Commission to the European Parliament, the Council, the European Economic and Social Committee and the Committee of the Regions, COVID-19 - Sustaining EU Preparedness and Response: Looking ahead, Available here https://health.ec.europa.eu/system/files/2022-04/covid-19_com_2022_190_en_0.pdf
